# Chemical Fingerprinting, Antioxidant, and Anti-Inflammatory Potential of Hydroethanolic Extract of *Trigonella foenum-graecum*

**DOI:** 10.3390/antiox11020364

**Published:** 2022-02-11

**Authors:** Hina Fatima, Muhammad Shahid, Chris Pruitt, Meredith A. Pung, Paul J. Mills, Muhammad Riaz, Rizwan Ashraf

**Affiliations:** 1 Department of Biochemistry, University of Agriculture, Faisalabad 38000, Pakistan; mshahiduaf@uaf.edu.pk; 2 Herbert Wertheim School of Public Health and Human Longevity Science, University of California, San Diego, CA 92093, USA; cpruitt@health.ucsd.edu (C.P.); mpung@health.ucsd.edu (M.A.P.); 3 Faculty of Life Sciences, University of Central Punjab, Lahore 54000, Pakistan; 4 Department of Allied Health Sciences, Sargodha Medical College, University of Sargodha, Sargodha 40100, Pakistan; riaz.elahi@uos.edu.pk; 5 Department of Chemistry, University of Agriculture Faisalabad, Faisalabad 38000, Pakistan; chemist1.dtlfsd@punjab.gov.pk

**Keywords:** *T. foenum-graecum*, air pouch inflammation, antioxidants, oxidative stress markers, cellular infiltration, lipid peroxidation, peritonitis

## Abstract

In the current study, the antioxidant and anti-inflammatory potential of hydroethanolic extract of *T. foenum-graecum* seeds was evaluated. Phenolic profiling of *T. foenum-graecum* was conducted through high-performance liquid chromatography-photodiode array (HPLC-PDA) as well as through the mass spectrometry technique to characterize compounds responsible for bioactivity, which confirmed almost 18 compounds, 13 of which were quantified through a chromatographic assay. In vitro antioxidant analysis of the extract exhibited substantial antioxidant activities with the lowest IC_50_ value of both DPPH (2,2-diphenyl-1-picrylhydrazyl) and ABTS (2,2′-azino-bis-3-ethylbenzothiazoline-6-sulfonic acid) inhibition assays. The extract was found to be non-toxic against human RBCs and murine macrophage RAW 264.7 cells. Moreover, the extract significantly (*p* < 0.001) reduced the lipopolysaccharide (LPS)-induced tumor necrosis factor alpha (TNF-α), intrlukin-6 (IL-6), prostaglandin E2 (PGE2), and nitric oxide (NO) in RAW 264.7 cells in a concentration-dependent manner. The hydroethanolic extract of *T. foenum-graecum* exhibited considerable anti-inflammatory potential by decreasing the cellular infiltration to the inflammatory site in both carrageenan-induced peritonitis and an air pouch model of inflammation. Pretreatment with *T. foenum-graecum* extract caused significant improvement in antioxidants such as superoxide dismutase (SOD), CAT (catalase), malondialdehyde (MDA), and myeloperoxidase (MPO) against oxidative stress induced by carrageenan. Based on our results of in vivo and in vitro experimentation, we concluded that hydroethanolic extract of *T. foenum-graecum* is a potential source of phenolic compounds with antioxidant and anti-inflammatory potential.

## 1. Introduction

Inflammation is the protective response of the body to noxious stimuli, microbes, and chemicals or irritants [1]. It causes change in vascular permeability, blood flow alteration and increased migration of leucocytes to the inflammatory area, and results in pain, heat, redness, swelling and functional failure of the affected tissue [2]. Several pathological processes, including arthritis, diabetes, cancer, and other severe inflammatory conditions, are usually characterized by pain and inflammation [3]. Although several antioxidant, antinociceptive, and anti-inflammatory medicines are available, these drugs are arguably inaccessible, costly, less effective, and have multiple side effects [4]. Non-conventional medicines occupy a significant place in healthcare systems, as more than 80% of people worldwide depend on them for their daily healthcare requirements, particularly in Asia and Africa [5].

*Trigonella foenum-graecum* (Fenugreek) is a valuable medicinal plant belonging to the Fabaceae family [6]. Plant seeds are mostly used in Asian, African, and Mediterranean countries as major ingredients of daily diets and in domains such as cosmetics, fragrances, beverages, nutrition, medicine and industry [7]. The major pharmacological attributes of fenugreek are hypotensive, antioxidant, antiviral, anticarcinogenic, galactagogue, laxative, febrifuge, carminative, anticholesterolemic, antimicrobial, etc. [8,9]. Overproduction of nitrogen and reactive oxygen species, as well as insufficient quenching/stabilization in the body, causes oxidative stress, which destroys important biomolecules (nucleic acids, lipids, and proteins) [10]. Several human diseases, including cardiac disorders, diabetes, cancer, inflammation, and neurodegenerative diseases, are exacerbated or triggered by oxidative damage to these biological molecules [11]. Plant-derived natural products, particularly polyphenolics, and related antioxidant phytochemicals have a diverse range of bioactivities, including anti-inflammation, owing to their ability to quench and alleviate oxidative stress in biological systems, thus restoring health [12]. As a result, the latest research focus has switched towards plant-based natural products as one of the most encouraging sources of curative agents of inflammation and pain [13]. 

Plants are an abundant source of bioactive compounds, including phenolics, phenolic acids, simple phenolics, flavonoids, derivatives of hydroxycinnamic acid, and anthocyanins [14]. Based upon their physiological properties, including scavenging of free radicals, as well as antimutagenic, anti-inflammatory, and anti-carcinogenic effects, phenolic compounds of different classes continue to attract much scientific attention [15]. Previously, anti-inflammatory properties of *T. foenum-graecum* have been reported against the carrageenan-induced paw edema model of inflammation, but there is no report available against carrageenan-induced air pouch inflammation and carrageenan-induced peritonitis. The antioxidant effects of polyphenolics are mainly due to their redox potential, serving as hydrogen donors, potent reducing agents, metal chelators, along with singlet oxygen quenchers [16,17,18]. Several phenolics and flavonoids have been demonstrated to have significant effects on the functioning of the immune system, including inflammatory processes [19]. Quercetin, luteolin, hesperidin, and apigenin are flavonoids containing potential anti-inflammatory activities [20]. Therefore, the current work was planned to investigate the in vitro antioxidant profiling and anti-inflammatory potential of hydroethanolic extract of *T. foenum-graecum* as a potential source of a safe, effective, available, and cheap antioxidant and anti-inflammatory therapeutic agent. The plant was selected due to an ethnomedical record of its use in Ayurvedic medicine in managing oxidative-stress-linked illnesses such as diabetes, as well as hypolipidemic, antihypertensive, and reproductive disorders such as the treatment of hormonal disorders, inducing labor, increasing milk supply and decreasing menstrual pain.

## 2. Materials and Methods

### 2.1. Collection of Plant Material, Identification, and Extract Preparation

*T. foenum-graecum* seeds were collected from market and identified by botanists at the Department of Botany, University of Agriculture, Faisalabad, Pakistan. Seeds were washed, shade dried and grinded with electric mill. Bioactive compounds from *T. foenum-graecum* seed powder were extracted though solvent (ethanol: water mixture (7:3)) under constant shaking for 72 h. The process was repeated thrice. The sample mixture was collected, filtered, and concentrated on rotary evaporator. The dried seed extract was re-suspended in dimethyl sulfoxide (DMSO) to obtain different concentrations. The extracts were kept in refrigerator until used [21].

### 2.2. Chemical Fingerprinting of T. foenum-graecum Seed Extract

#### 2.2.1. HPLC-PDA Analysis

The analytical and instrumental parameters, i.e., mobile phase composition, flow rate, and temperature, were optimized to achieve good separation among the phenolic profile of *T. foenum-graecum* extract by following the protocol of Hasany et al. [22]. The high-efficiency reverse-phase octadecyl column Spherisorb ODS-2 (Waters Corporation, Milford, MA, USA) bearing a particle size of 10 μm and dimensions (length × internal diameter) of 300 mm × 4.6 mm was used under gradient mode of elution on high-performance liquid chromatography equipped with photo diode array detector (HPLC-PDA) (waters alliance 2998). For the 0.5% acetic acid in water (A), when mixed with organic solvent methanol (B) in the sequence 80A:20B (0–3 min), 70A:30B (3–6 min), 65A:35B (6–9 min), and 55A:45B (10–20 min) and run in the mobile phase at 1 mL/min, the well-resolved peaks were observed within 40 min analysis time.

#### 2.2.2. LC-MS/MS Analysis

UHPCL equipped with mass detector (QTOF-MS/MS) Agilent 6520 was used for the analysis of sample. UHPLC chromatographic conditions were used as mobile phase in gradient mode of elution: (A) 0.1% formic acid in water and (B) 0.1% formic acid in methanol and gradient flow with 10–20% A at 1–10 min, 20–30% A at 10.1-20 min, 30–50% A at 20.1–30 min, and 50–10% A at 30.1–40 min while flow rate was set at 0.5 mL/min. Column used was octadesylsilane waters (4.6 × 100 mm, 2.5 µm) at ambient temperature. Ionization source was ESI (electrospray ionization) source operating at both positive and negative ion modes. In the ionization source, pure nitrogen gas was used as collision as well as drying gas. The capillary temperature was adjusted to 350 °C and nebulizer pressure was set to 35 psi. Ion source parameters, including flow rate of drying gas was maintained to 10 L/min, while VCap, octapole RF peak voltages, fragmentor, and skimmer were maintained to 3500, 740, 150, and 65 V, respectively, and mass range was 150–1200 Da. MS/MS fragmentation was acquired at selected precursor ions of each peak [23,24].

### 2.3. Determination of Total Phenolic and Total Flavonoid Contents

Total phenolic contents in *T. foenum-graecum* seed extract were examined by the spectrophotometer method adopted by Liu et al. [25]. Briefly, *T. foenum-graecum* extract (20 µL) was mixed with Folin–Ciocalteu reagent (100 µL), distilled water (1.16 mL), and sodium carbonate solution (200 µL, 20%) and incubated for 30 min at 40 °C. Absorbances of reaction mixture were measured at 700 nm. TPCs in plant extract were calculated from calibration curve prepared by different concentration of gallic acid, and results are given as mg of gallic acid equivalents (GAE)/gram of plant dry weight. For TFC, plant extract (100 µL), deionized water (200 µL), sodium nitrite (250 µL, 5%), and aluminum chloride (250 µL, 10%) were mixed and reaction mixtures were incubated for 6 min. After incubation, sodium hydroxide solution (2.5 mL, 1 M) was added and incubated for another 15 min. The reaction mixture was then diluted with deionized water (2.5 mL) and read the absorbance at 500 nm. TFC in seed extracts were calculated from the calibration curve of catechin as CE (catechin equivalents)/microgram of dry weight of plant extract.

### 2.4. Measurement of Total Antioxidant Capacity (TAC)

For quantification of TAC, ammonium molybdate reagent (0.004 M ammonium molybdate, 0.028 M sodium phosphate, 0.6 M sulphuric acid) was prepared. *T. foenum-graecum* seed extract (0.1 mL) was mixed with 1 mL of ammonium molybdate reagent and 28 μL of sodium nitrite (5%). The reaction mixtures were heated in water bath at 95 °C for 90 min. The reaction mixtures were cooled and absorbances of reaction mixtures were noted at 765 nm against blank containing ammonium molybdate reagent only. TAC in plant extract was calculated in milligrams of ascorbic acid equivalents per gram of plant dry weight (mg AAEg−1DW) [26].

### 2.5. In Vitro Antioxidant Profiling of T. foenum-graecum Seed Extract

#### 2.5.1. ABTS Cation Inhibition Assay

The ABTS cation radicals were generated by mixing stock solution of ABTS (7 mM, 5 mL) with potassium persulfate (148 mM, 88 µL), and diluted with sodium acetate buffer (pH 4.5, 20 mM) having optical density of 0.70  ±  0.02 at 734 nm. Plant extract (12 μL) was mixed with ABTS solution (188 μL) and kept for 30 min at RT. Reaction mixtures were noted at 734 nm [27].

#### 2.5.2. DPPH Inhibition Assay

The DPPH free radicals were formed by mixing DPPH (8.87 mM) with methanol (99.9%). After which 10 µL of plant extract and 190 μL of DPPH solution was mixed and incubated at 30 °C for 15 min. Absorbances were noted at 520 nm [28].

### 2.6. In Vitro Cytotoxicity of T. foenum-graecum Seed Extract

#### 2.6.1. Hemolytic Assay

Hemolytic activity of *T. foenum-graecum* seed extract was performed against human RBCs [29]. For this, fresh human blood (5 mL) was centrifuged for 5 min at 4000 rpm. Next, supernatant was drained, and cells were washed three times with phosphate buffer saline and 10% RBC suspension was prepared. *T. foenum-graecum* seed extract (20 µL) was mixed with 180 µL of RBC suspension in sterile tubes and mixed with agitation. The reaction mixtures were centrifuged (5000 rpm, 5 min) after incubation of 30 min at room temperature. Supernatant (100 µL) was mixed with PBS (900 µL). Triton-X (0.1%) and PBS were used as controls. Absorbances of reaction mixtures were noted at 576 nm.

#### 2.6.2. Thrombolytic Activity

Thrombolytic activity of *T. foenum-graecum* seed extract was performed by clot lysis method [30]. The clot lysis (%) activity was measured against streptokinase by using the following formula:

Thrombolytic activity (%) = (Wc1 − Wc2)/Wc1 × 100

Wc1 and Wc2 represent clot weights before and after lysis, respectively.

### 2.7. Cell Culture

The RAW 264.7 cells were provided by the Department of Cellular and Molecular Medicine, University of California, San Diego, CA, USA, and grown in DMEM (Dulbecco’s Modified Eagle Medium), 10% of FBS (fetal bovine serum) and streptomycin (100 μg/mL), and penicillin (100 units/mL) solution and incubated at 37 °C and 5% CO_2_ under humified conditions. Cells were counted by Trypan blue exclusion method [31].

#### 2.7.1. Cytotoxicity Determination

For cytotoxicity determination of *T. foenum-graecum* seed extract, RAW 264.7 cells (2 × 105) were seeded in 96-well plate overnight at 37 °C under humified conditions at 5% CO_2_. The next day, different concentrations of *T. foenum-graecum* (100–1000 µL) were added and cells were again incubated at 37 °C for 72 h at 5% CO_2_. Doxorubicin was used as positive control. After incubation, 20 µL of MTT solution (5 mg/mL) was added into each well and further incubated for another 4 h. Then, microplate was centrifuged at 800× *g* for 20 min, supernatant was drained, DMSO (100 µL) was added to dissolve formazan crystals, and signal was recorded at 570 nm [32].

#### 2.7.2. Enzyme-Linked Immunosorbent Assay (ELISA)

RAW 264.7 cells (2 × 10^5^) were grown overnight in 96-well microplate and treated next day with different concentrations (50–300 µg/mL) of *T. foenum-graecum* extract and standard dexamethasone (10–60 µg/mL) and stimulated with LPS (1 µg/mL). Cells were incubated for 24 h at 5% CO_2_. After incubation, cells were centrifuged and supernatant was stored at −80 °C for TNF-α and IL-6 analysis [33,34].

#### 2.7.3. Measurement of NO and PGE_2_

For measurement of NO and PGE_2_, RAW264.7 cells were (2 × 10^5^) seeded in DMEM medium with 10% of FBS, streptomycin (100 μg/mL) and penicillin (100 units/mL) solution for 24 h, and next different concentrations of *T. foenum-graecum* (50–300 µg/mL) and standard dexamethasone (10–60 µg/mL) were added, and cells were stimulated with LPS (1 µg/mL) for 24 h at 5% CO_2_. After 24 h, plate was centrifuged, and supernatant was collected and analyzed for NO and PGE_2_ [35,36].

### 2.8. In Vivo Toxicological Evaluation of T. foenum-graecum Seed Extract

#### 2.8.1. Ethical Considerations

In vivo animal study was approved by Institutional Animal Care and Use Committee (IACUC), University of Agriculture Faisalabad, Pakistan.

#### 2.8.2. Animals

Healthy albino rats (150–200 g) were kept at 24 °C in polypropylene cages provided with softwood shavings as bedding material, with standard conditions and free access to standard rodent diet with water ad libitum. Prior to dosing, all rats were adapted to the laboratory setting for a time period of one week [37].

#### 2.8.3. Acute and Subacute Toxicity Analysis

The rats were assigned into two groups, Group I labelled as placebo control receiving saline solution and Group II as the treatment group received dose of 2000 mg/kg BW *T. foenum-graecum* extract at single. The treatment was given only once on starting day of experimentation and rats were observed for behavioral motor and neuronal activities, including sleep, salivation, eye color, convulsions, lethargy, skin and fur appearance, tremors, and diarrhea. All activities were monitored closely and recorded at different time intervals. In the absence of symptoms of toxicity or mortality during acute toxicity period, subacute toxicity analysis was performed on new set of rats randomly assigned into four different groups and human equivalent doses (250, 500 and 1000 mg/kg BW, respectively) were given daily for 28 days. All experiments were carried out by using OECD (Organization of Economic Co-operation and Development) guidelines and rats were monitored in the same way as in acute toxicity. Animals were euthanized after 28 days, and blood samples, and parts of different organs were stored for hematological, biochemical, and histopathological profiling. The animals were properly disposed of in accordance with the established procedures [38].

### 2.9. Anti-Inflammatory Potential

#### 2.9.1. Air Pouch Inflammation

Air pouch model of inflammation was used for assessment of in vivo anti-inflammatory potential of *T. foenum-graecum* seed extract [39]. Animals were randomly assigned into six groups. Air pouch on intracapsular region of rats was generated by injecting 5 mL of sterile air into dorsal side. An additional 3 mL of air was injected to air pouch after three days. After seven days of first injection, a carrageenan solution (0.5 mL, 1.5%) was given into the air cavity directly to execute inflammatory response. *T. foenum-graecum* seed extract treatment (100, 200 and 400 mg/kg) was also given along with carrageenan into the air cavity directly. Animals were sacrificed at different time points (6, 12, 24 h) and through cervical dislocation and pouch tissue were dissected precisely to collect the inflammatory exudate. Cellular infiltration in inflammatory exudate was measured to assess the anti-inflammatory response of plant extract. Morphological changes in the pouch tissues were also observed through histopathological examination.

#### 2.9.2. Carrageenan-Induced Peritonitis

Animals (randomly assigned into six groups) were pretreated orally with *T. foenum-graecum* seed extract (100, 200, 400 mg/kg BW), saline solution (0.9%, placebo control) and dexamethasone (20 mg/kg BW, standard group) before 30 min of intraperitoneal injection of carrageenan. Then, animals were slaughtered after 4 h of carrageenan injection, and peritoneal cavities of animals were washed with normal saline solution. Cellular count was performed in peritoneal fluid by dissolving 20 µL of peritoneal fluid in Turk’s solution (0.38 mL). The collected peritoneal fluid was centrifuged at 10,000 rpm for 10 min and stored at −8 °C for analysis of oxidative stress and lipid per-oxidation parameters [40].

#### 2.9.3. Measurement of Oxidative Stress Parameters and Lipid Peroxidation

Oxidative stress markers, including total oxidant status (TOS), total antioxidant status (TAS), and lipid peroxidation markers, i.e., myeloperoxidase (MPO) and malondialdehyde (MDA), along with superoxide dismutase (SOD) and catalase (CAT) level in air pouch, exudate and peritoneal fluid, were also measured [41,42,43,44,45].

### 2.10. Statistical Analysis

To determine statistical significance, one-way ANOVA was performed followed by multiple comparison tests through Tukey’s test. Obtained data were presented as mean ± standard deviation of the mean. IC_50_ values were also calculated using regression analysis. All analysis were performed using GraphPad Prism version 8 software (Graphpad Software Inc., San Diego, CA, USA) [46].

## 3. Results

### 3.1. Screening of Phytochemicals through HPLC-DAD

**Figure 1:**
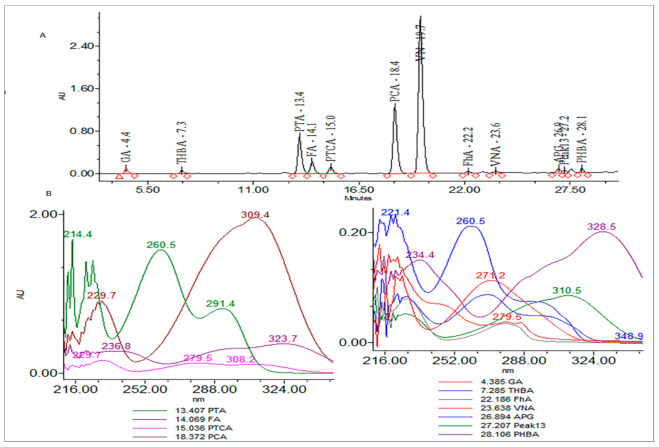
(**A**) Chromatogram of high-pressure liquid chromatography equipped with diode array detector (HPLC-DAD) at 280 nm and (**B**) extracted spectrum (200–400 nm) of each peak.

**Figure 2:**
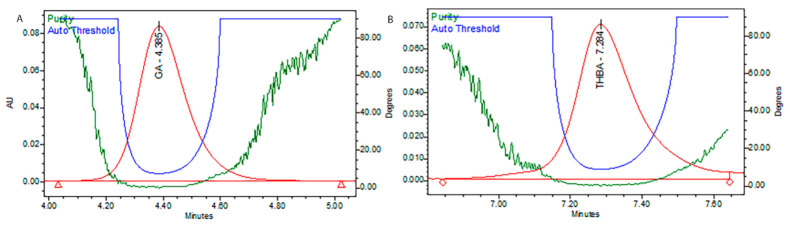
Purity plots of representative peaks, (**A**) gallic acid, and (**B**) trihydroxybenzoic acid.

*T. foenum* extract was characterized for its bioactive constituents through HPLC-DAD using operating conditions previously discussed in the materials and method section (Figure 1). During the analysis, 13 compounds were identified in plant extract that were mostly phenolic acids and flavones. Peak 1 was identified as gallic acid having a response intensity of 0.17 AU (absorption unit), provided in Figure 1. When this peak was extracted for the PDA (photodiode array detector) spectrum, it showed lambda maximum (λ_max_ nm) at 271.2 and 214.6 nm, which corresponds to a standard spectrum as well as the NIST library. The purity of the peak was also assured through the 3D spectrum as well as measuring the purity angle and purity threshold of the peak, which are given in Figure 2 as well as Table 1. The concentration of gallic acid was measured by comparing the area under the peak in comparison to the area under the peak of standard and determined 117.6 ± 1.5 mg/100 g DW. The other peaks that appeared in the chromatogram of the sample were investigated for their identification and quantification by comparison with the standards run as well as the NIST library, and the results are summarized in Table 1. The most abundant antioxidant and antimicrobial compound found was p-coumaric acid with a concentration of 256.7 ± 6.8 g/100 g of DE followed by ferulic acid 168.4 ± 1.8 g/100 g of DE. The results were in agreement with previous reports by [47,48], who reported the abundance of p-coumaric acid in plant extracts. The identification of phytochemicals indicates that antioxidant and other biological properties of extract could be due to the presence of these bioactive compounds. Almost all the phenolic acids identified have a hydroxyl group that could be responsible for the scavenging potential of these compounds [49]. Peaks of the chromatogram were identified through comparison with standards as well as the match index of the UV-visible spectrum of each peak using the NIST library. The identified compounds were presented in order of their elution on the reverse phase column.

### 3.2. UHPLC-Q-TOF Chromatogram of T. foenum

**Figure 3:**
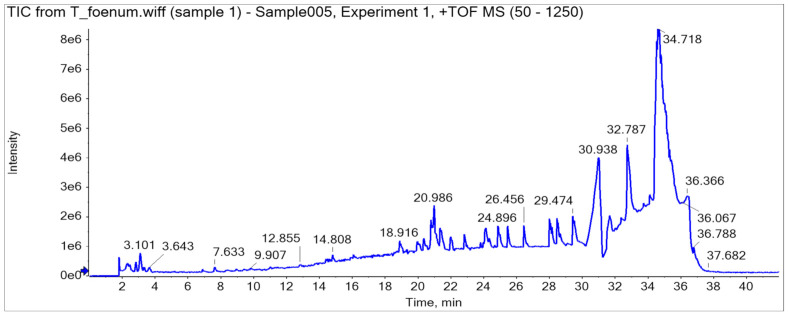
UHPLC-Q-TOF chromatogram of *T. foenum*.

HPLC analysis of plant extract led to the identification and quantification of 13 phenolics, and samples were further characterized through LC-MS/MS (Q-TOF) profile (a detailed description is provided in Table 2, Figure 3). A total ion current (TIC)-based chromatogram of plant extract of *T. foenum* is presented in Figure 2, and the precursor ion of each peak was further processed for fragmentation pattern (MS/MS) and compared with the NIST library as well as literature reported for the identification of phytochemicals present in the sample. The results are summarized in Table 2, which described each component with retention time in order of their elution order and fragmentation pattern. Although numerous peaks were recorded in the chromatogram, we reported almost 18 compounds, which were identified through the NIST library or literature. MS profiling of peak 1 (RT 2.34 min) produced a parent ion peak at [M *−* H]+ *m*/*z* 441.0840 Da and daughter ions 251.0365, 233.0297 and 124.9848 Da, which corresponds to catechin gallate, and its fragmentation pattern revealed it would be catechin 3-O-gallate. MS spectra are provided in Supplementary Materials Figure S1. The other peaks appeared in chromatogram were also processed for MS1 as well as MS2 at both positive and negative ion mode, and the results were compared with literature and are described in Table 2. Almost 18 compounds were confirmed through literature and their detailed description is provided in Supplementary Materials Figures S1–S17.

### 3.3. Determination of TPC, TFC and In Vitro Antioxidant Activities

The results of the in vitro antioxidant profiling of *T. foenum-graecum* seed extract are presented in Table 3. The results reveal that *T. foenum-graecum* seed extract displayed excellent antioxidant potential with total phenolic contents of 454.93 ± 3.57 mg GAE/g, total flavonoid contents (TFC) of 135.04 ± 2.12 µg/CE and total antioxidant capacity (TAC) of 162.51 ± 3.81 per gram of dry plant extract. The extract represented a concentration-dependent activity of DPPH inhibition and ABTS scavenging assay with an IC_50_ value of 24.7 ± 2.70 and 15.8 ± 0.87 µg/mL, respectively, which is similar to standard.

### 3.4. Toxicological Analysis of T. foenum-graecum Seed Extract

**Figure 4:**
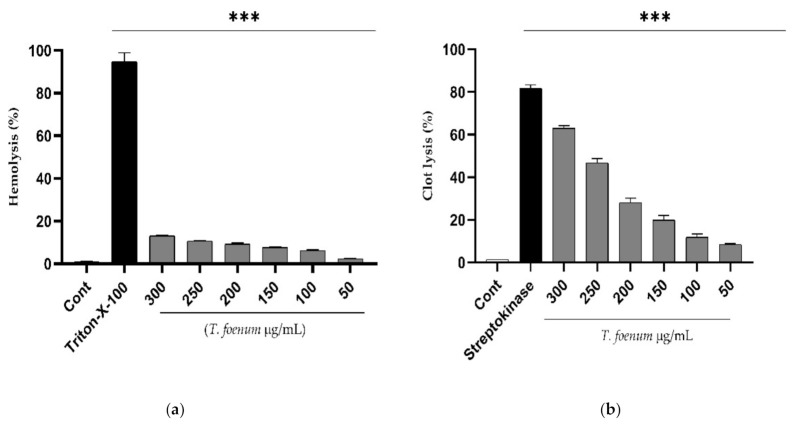
Hemolytic (**a**) and thrombolytic activity (**b**) of different doses (50–300 µg/mL) of hydroethanolic extract of *T. foenum-graecum* seeds through hemolytic and thrombolytic assays. (***, significant at *p* < 0.001).

The results of hemolytic activity show that *T. foenum-graecum* seed extract has negligible toxicity in comparison to Triton-X (positive control), as shown in Figure 4. Further, the extract exhibits dose-dependent activity, and the hemolysis of erythrocytes increased with an increase in concentration of *T. foenum-graecum* seed extract. The concentration required for hemolysis of 50% RBCs (HC_50_ values) was estimated to be 2838 µg/mL, which is significantly (*p* < 0.001) different from HC_50_ of Triton-X-100 (64.98 µg/mL). Similarly, *T. foenum-graecum* extract showed a dose-dependent increase in clot lysis activity. Standard streptokinase (0.5 mL) and *T. foenum-graecum* extract at a high concentration (300 µg/mL) showed significant (*p* < 0.001) clot lysis activity of 81.82% and 63.01%, respectively comparing with the negative control (1.36%).

### 3.5. Cytotoxicity Determination

**Figure 5:**
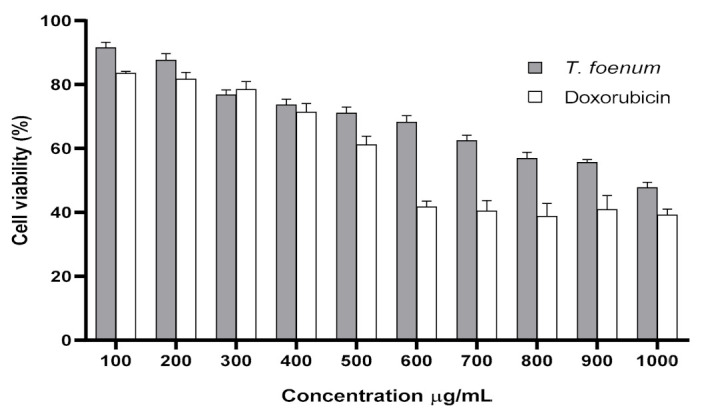
Cytotoxic activity (cell viability) of hydroethanolic extract of *T. foenum-graecum* and doxorubicin (standard) against RAW 264.7 macrophage through MTT assay.

The results of cytotoxicity of *T. foenum-graecum* seed extract against RAW 264.7 cells are presented in Figure 5. Increased concentration of *T. foenum-graecum* seed extract has a negative impact on cell viability. The IC_50_ values for *T. foenum-graecum* seed extract and standard doxorubicin against RAW 264.7 cells were 1055 and 614.9 μg/mL, respectively. The results exhibit that plant extract showed low-to-moderate cytotoxicity in a concentration-dependent manner. Cell viability percentage was decreased with an increase in the concentration of plant extract. Cell viabilities in murine macrophages incubated with a different concentration (100–1000 µg/mL) of *T. foenum-graecum* seed extract were 91.53%, 87.64%, 76.79%, 73.67%, 71.06%, 68.25%, 62.47%, 56.87%, 55.60% and 47.78%, respectively. At concentrations (100–300 μg/mL), little cytotoxic effects were observed, and these concentrations were adopted further to examine the anti-inflammatory activity of *T. foenum-graecum* seed extract.

### 3.6. Effect of T. foenum-graecum Extract on TNF-α and IL-6

The tested concentration (50–300 μg/mL) of *T. foenum-graecum* seed extract showed a substantial decrease in the production of IL-6 (1889.92 ± 19.32, 1338.73 ± 12.14, 1330.42 ± 10.56, 1108.32 ± 20.87, 947.33 ± 16.45, 748.52 ± 9.45, respectively) (Figure 4a and TNF-α (2158.12 ± 31.87, 1715.21 ± 28.68, 1518.68 ± 26.98, 1045.01 ± 23.55, 741.33 ± 18.84, 522.99 ± 19.03, respectively) (Figure 4b) in comparison to LPS-stimulated macrophages (2177.83 ± 37.56 µg/mL for TNF-α and 3894.42 ± 49.73 pg/mL for IL-6), suggesting significant in vitro anti-inflammatory potential. Figure 4a,b, demonstrates the effect of *T. foenum-graecum* seed extract on the production of TNF-α and IL-6 in RAW 264.7 cells after stimulation with LPS at various concentrations. The results indicate that the plant extract inhibited both TNF-α and IL-6 production significantly (*p* < 0.001) at different concentrations of 50–300 μg/mL, with inhibition rates of 13.21%, 38.91%, 38.52%, 49.10%, 56.50%, 65.62% and 44.58%, 55.95%, 61.03%, 73.16%, 80.96%, 86.57%, respectively, with IC_50_ values of 192.7 µg/mL and 72.03 µg/mL.

### 3.7. NO and PGE2 Quantification

**Figure 6:**
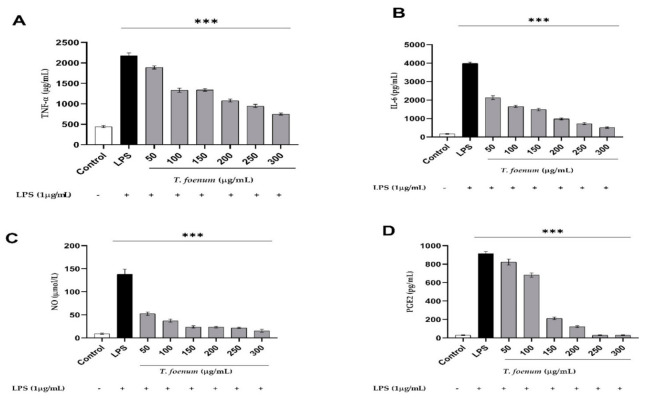
Anti-inflammatory profile of *T. foenum-graecum* seed extract through quantification of, TNF-α (**A**), IL-6 (**B**), NO (**C**) and PGE2 (**D**) measurement in RAW 264.7 cell’s culture medium after stimulation with LPS (1 µg/mL). +, treated with LPS, ***, significant at *p* < 0.001.

The results of NO and PGE2 inhibition by *T. foenum-graecum* seed extract are presented in Figure 6C,D. The findings show that both NO and PGE2 are significantly (*p* < 0.001) inhibited by *T. foenum-graecum* extract in culture of RAW 264.7 cells, reaching to the level of the control (without LPS) at higher concentrations of plant extract in a dose-dependent manner (Figure 6C,D). Moreover, a significant (*p*  <  0.0001) increase in NO and PGE2 concentration was recorded after treatment with LPS, which was markedly (*p*  <  0.001) restored to the normal level after treatment with standard and plant extract with IC_50_ values of 11.40 and 122.0 µg/mL.

### 3.8. In Vivo Assays

#### 3.8.1. Acute and Subacute Toxicity Analysis of *T. foenum-graecum* Seed Extract

The results from the acute toxicity analysis show that *T. foenum-graecum* seed extract did not show any harmful effects in the treated animals, as compared with the control group at a dosage of 2000 mg/kg BW. No morbidity or mortality was noticed in experimental animals during acute toxicity analysis. Consequently, the LD_50_ value of *T. foenum-graecum* seed extract examined was calculated to be >2000 mg/kg BW. During acute toxicity analysis, none of the animals exhibited any changes in behavior or symptoms related to the circulatory, respiratory, central, and autonomic nervous system (Table 4). As *T. foenum-graecum* extract treatment did not exhibit any adverse effects in the acute study, a human equivalent dose was selected for the long-term subacute toxicity study of 28 days. In subacute toxicity analysis, the effect of plant treatment on weight, relative organ weight, liver and kidney function indices, and hematological and histopathological parameters was evaluated. Table 5 shows the variation in body weight of rats after treatment with different doses of *T. foenum-graecum* seed extract. The body weight of animals treated with plant extract at different dosages (250, 500, and 1000 mg/kg) did not differ significantly (*p* > 0.05) from those in the control group. Similarly, in comparison to the control group, there was no significant (*p* > 0.05) difference in the absolute weights of the kidney and liver of treated animals (Table 6).

#### 3.8.2. Hematological and Biochemical Indices

The results of the subacute toxicity of *T. foenum-graecum* seed extract treatment on hematological parameters are presented in Table 7, and findings reveal that hematological parameters of treatment animals were not affected after treatment with plant extract for 28 days. Similarly, treatment of *T. foenum-graecum* seed extract in subacute toxicity did not cause any significant change in liver and kidney function indices (Table 8). No significant alteration in AST, ALT, ALP, γ-GT, total proteins, bilirubin, glucose, creatinine, total cholesterol, total glycerides, or blood urea nitrogen was detected in experimental animals in comparison to the control group. The little variation represented has no clinical significance, because values recorded are within the normal range of rats. Histopathological examinations of liver and kidney tissues did not exhibit any morphological alterations or abnormalities under the light microscope.

### 3.9. In Vivo Anti-Inflammatory Potential

#### 3.9.1. Air Pouch Model of Inflammation

**Figure 7:**
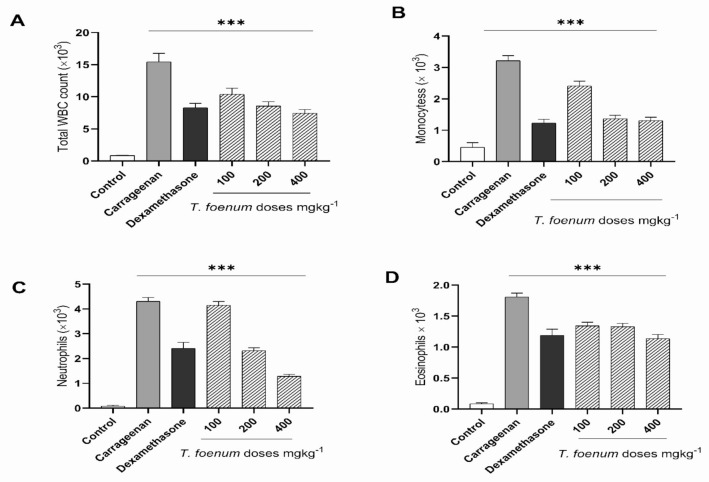
Anti-inflammatory profile (cellular infiltration) (**A**) (Total WBCs), (**B**) (Monocytes), (**C**) (Neutrophils) and Eosinophils (**D**) of hydroethanolic extract of *T. foenum-graecum* against air pouch model of inflammation. Data are expressed as mean ± S.D. ***, significant at *p* < 0.001.

**Figure 8:**
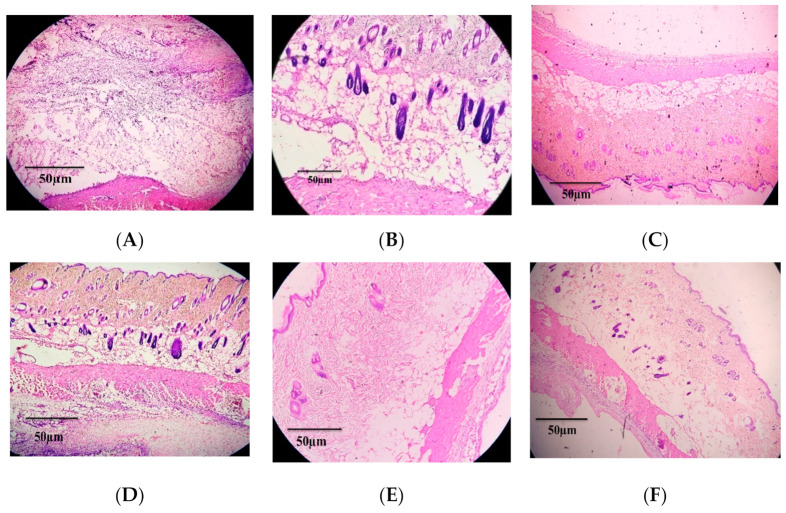
Histopathological photographs of air pouch tissues after treatment with different concentrations of *T. foenum-graecum* extract at 100, 200, 400 mg/kg BW (**A***–***C**), carrageenan control (**D**), dexamethasone 20 mg/kg body weight (**E**) and normal control (**F**).

To evaluate the anti-inflammatory potential of *T. foenum-graecum* seed extract, inflammation of air pouch model was utilized, and inflammatory exudate was analyzed for different blood cells associated with inflammation (Figure 7). In inflammatory exudate of rats treated with carrageenan only, white blood cell (15 × 10^3^ cells/mL) count was about 20-fold higher compared to the control group (0.83 × 10^3^ cells/mL). Dexamethasone (10 mg/kg BW) treatment caused an almost 10-fold reduction (8.13 × 10^3^ cells/mL) in WBC count. *T. foenum-graecum* seed extract suppressed the WBC population dose-dependently. For example, WBC count in the 200 mg/kg BW treatment group was equal to dexamethasone, and in the 400 mg/kg BW treatment group the effect was more significant than dexamethasone. As shown in Figure 5, injection of carrageenan caused a noticeable increase in monocytes as compared to the carrageenan control group. However, *T. foenum-graecum* extract by doses of 200 and 400 mg/kg BW led to significant (*p* < 0.001) reductions in monocytes (1.37 × 10^3^ and 1.31 × 10^3^, respectively) as compared to the carrageenan control (3.21 × 10^3^). Moreover, *T. foenum-graecum* extract significantly reduced the carrageenan-induced proliferation of eosinophils and neutrophil in treatment groups, as compared to carrageenan-treated animals, in a dose-dependent manner, and this effect is comparable to dexamethasone (20 mg/kg BW), particularly at 400 mg/kg BW of *T. foenum-graecum*. Similarly, histopathological examination of air pouch tissue also exhibited a change in the thickness of the air pouch membrane and marked cellular influx to the inflammatory site with increased inflammatory response. The treatment with *T. foenum-graecum* extract and dexamethasone caused an increase in membrane thickness as compared to the carrageenan-treated group, in which the membrane was narrow and condensed and cellular influx was also reduced (Figure 8).

#### 3.9.2. Carrageenan-Induced Peritonitis

**Figure 9:**
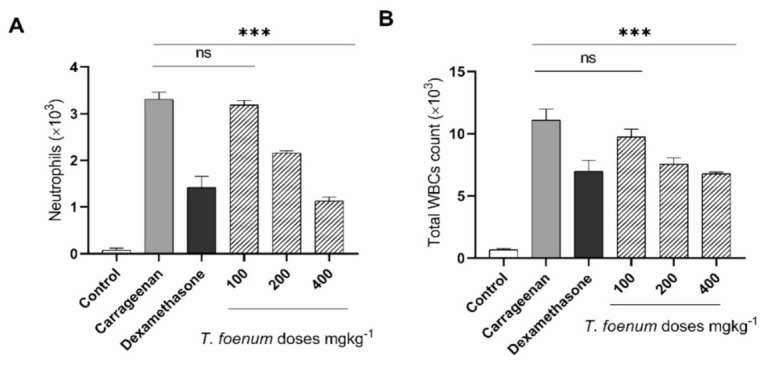
Anti-inflammatory effect A (Neutrophils) and B (Total WBCs) of hydroethanolic extract of *T. foenum-graecum* on carrageenan-induced peritonitis. Rats were pretreated orally with different concentrations of *T. foenum-graecum* extract and dexamethasone (20 mg/kg body weight). Data are expressed as mean ± SD. ***, significant at *p* < 0.001.

Treatment of *T. foenum-graecum* seed extract before 30 min of intraperitoneal injection of carrageenan significantly (*p* < 0.001) reduced total WBC and neutrophil count in peritoneal exudate (Figure 9). Injection of carrageenan caused a 16-fold increase in WBC count (11.12 × 10^3^) as compared to the control (0.66 × 10^3^), and dexamethasone and *T. foenum-graecum* (200 and 400 mg/kg dose) decreased it to 6.97 × 10^3^. Moreover, *T. foenum-graecum* extract significantly reduced the neutrophil count in peritoneal exudate of treated rats as compared to the control group.

#### 3.9.3. Effect of *T. foenum-graecum* on Antioxidant Enzymes and Stress Markers

The levels of enzymatic antioxidants, lipid peroxidation and oxidative stress markers in air pouch exudates and peritoneal fluid were significantly (*p* < 0.001) altered in the carrageenan-treated group as compared to the control group. However, treatment with dexamethasone and *T. foenum-graecum* significantly reversed the carrageenan-induced changes in these parameters. MPO and MDA levels in the carrageenan-treated group were significantly higher as compared to those of the control group (Table 9 and Table 10). Dexamethasone and *T. foenum-graecum* extract effectively reduced the MDA and MPO levels in the air pouch and peritoneal exudate, and the effect was dose dependent, with 400 mg/kg BW recorded as most effective dose. Similarly, carrageenan treatment increased the level of superoxide dismutase and catalase and hence TAS significantly (*p* < 0.001) at the inflammatory site, while dexamethasone significantly reduced the level of these enzymes, and this effect was similar to the *T. foenum-graecum* group at 400 mg/kg BW dose treatment group. Furthermore, total oxidant level (TOS) was also significantly higher among the carrageenan treatment group in comparison to that of the control group, which is significantly (*p* < 0.001) decreased in both dexamethasone and *T. foenum-graecum* treatment groups. The effect at higher doses of *T. foenum-graecum* (400 mg/kg) was similar to the dexamethasone-treated group.

## 4. Discussion

The in vitro and in vivo antioxidant and anti-inflammatory properties of the hydroethanolic extract of *T. foenum-graecum* were studied using different methods, and the overall findings have been corroborated. *T. foenum-graecum* is an old medicinal herb that dates back to Egyptian times and has significant antipyretic, anti-inflammatory and anti-nociceptic properties. In the current study, the phytochemical screening of *T. foenum-graecum* extract exhibited a considerable amount of phenolics and flavonoids and showed significant antioxidant activity. The results of our antioxidant and polyphenolic analysis are largely in agreement with Kenny et al. [56], who reported total phenolic contents (106.316 ± 0.377 mg GAE/g), FRAP (77.352 ± 0.627) and DPPH (35.338 ± 0.908) mg of Trolox equivalents per gram of Fenugreek ethyl acetate extract. Previously, Akbari et al. [57] also reported the ABTS and DPPH assays with IC_50_ values of 161.3 ± 2.21 and 172.6 ± 3.1 µg/mL. Similarly, TFC and TPC, as well as the fenugreek, were also reported (14.417 ± 0.23 mg QE/g and 38.97 ± 0.34 mg GAE, respectively). 

Evaluating the toxicity profile of medicinal plants and plant-based products is typically a preliminary step for the screening of the therapeutic potential of plant-derived products [19,57]. Another approach for the evaluation of cytotoxicity is hemolytic assay, which is described by the subsequent release of hemoglobin after erythrocytes lysis [58]. The polyunsaturated fatty acids and hemoglobin mostly attack RBCs because of their redox-active oxygen transportation property [59]. Consequently, the oxidative process damages the proteins and lipids in the erythrocyte membrane during hemolysis [60]. This damage is associated with several other factors, including oxidative drugs, a high quantity of transition metals, radiation, insufficient antioxidant defense system and hemoglobinopathies [61]. Hemolysis of erythrocytes occurs when they are exposed to toxic natural or synthetic compounds. The HC_50_ of *T. foenum-graecum* extract is very high compared to Triton-X-100 and thus supports its application as a successful pharmaceutical drug in practice. The thrombolytic potential of *T. foenum-graecum* extract is an important finding, which may have implications in cardiovascular health, especially in atherothrombotic patients. These results are in agreement with Ktari et al. [62], which also reported cytotoxicity of *T. foenum-graecum* extract, and no hemolytic activity was reported against bovine RBCs.

Many of the classic experiments in the field of inflammation have been performed using murine macrophages [4,5]. The hydroethanolic extract of *T. foenum-graecum* demonstrated a noticeable inhibition of pro-inflammatory cytokines, NO and PGE2 after LPS stimulation. The activation of pro-inflammatory cytokines is among the most fundamental processes that occur during inflammatory pathways [63]. TNF-α is a potent pro-inflammatory cytokine produced by a variety of immunocompetent cells, such as neutrophils, dendritic cells, macrophages, and T helper cells, and is capable of attracting immune cells to the inflammatory site to initiate the inflammatory process [64]. The hydroethanolic extract of *T. foenum-graecum* (50–300 μg/mL) presented dose–response anti-inflammatory activity, since it showed greater TNF-α and IL-6 inhibition in the culture of RAW 264.7 cells after stimulation with LPS. TNF-α and IL-6 are measured as basic markers of pro-inflammatory processes produced by macrophages and have the ability to activate T cells [65]. As a result, inhibiting these cytokines is thought to be an efficient way to prevent and cure a variety of inflammatory disorders [66]. The hydroethanolic extract of *T. foenum-graecum* also inhibited NO and PGE2 production by RAW 264.7 cells and showed similar results to those of TNF-α and IL-6 in relation to the positive control dexamethasone, evidencing its antioxidant and anti-inflammatory action [67]. NO is a powerful radical that regulates the growth, function, and death of a variety of cell types involved in inflammatory and immunological responses. Excessive NO generation has been linked to the pathophysiology of oxidative damage and inflammation [68]. Previous inhibition of pro-inflammatory cytokines by *T. foenum-graecum* extract has also been reported in some studies [35]. The anti-inflammatory attributes of *T. foenum-graecum* may be due to the large number of polyphenolic substances that have been reported good anti-inflammatory agents in previous studies [19,20].

Oral administration of extracts is the most appropriate and economical method of drug delivery in animal models during toxicity analysis [69]. Moreover, acute oral toxicity analysis in the rat model can effectively predict human acute lethal dosages in clinical setups [70]. The body weight of animals is an important index for the determination of the toxicity of synthetic or natural compounds [71]. In the current study, there was no abnormal change in the weight of animals among the treatment group and control group up to a dose of 1000 mg/kg. Likewise, variation in organ weight is also a good indication for plant-induced abnormalities, which are commonly linked with treatment-related effects. There were no significant variations in the body or organ weight of animals after 28 days of treatment with hydroethanolic extracts of *T. foenum-graecum* seed extract. All of the animals showed normal weight gain, with no significant differences among control and treatment groups. The hematopoietic system in both animals and humans is extremely sensitive to toxic substances and acts as a key indicator of pathological and physiological condition [72]. In toxicological assessment, biochemical parameters are of prime significance due to their extreme sensitivity and ability to respond against changes induced by toxicological substances [29]. These biochemical markers have a significant role in the evaluation of toxicological changes induced by natural or chemical substances [73]. In the current study, none of the biochemical parameters showed significant (*p* > 0.05) changes from the untreated control group. Similarly, the histopathological examination of both liver and kidney tissues was also found to be morphologically normal. Previously, some studies also reported the acute and subacute toxicities of *T. foenum-graecum* extract, and no toxicological effects were reported on biochemical and hematological markers [74,75].

Carrageenan-induced inflammation in rats is a useful approach for testing natural products with potential anti-inflammatory activity and also for further elucidating their mechanism of action [76]. Carrageenan injection initiates an acute inflammatory response linked with hyperalgesia, usually classified by edema and increased response to mechanical and thermal stimuli [40]. The carrageenan-induced inflammation is linked with increased leukocytes migration, mainly neutrophils, and enhanced myeloperoxidase (MPO) activity [77]. Pro-inflammatory mediators such as MPO and NOx and proinflammatory cytokine levels (TNF-α and IL-6) can modulate the inflammatory response and can significantly alter the amplitude of leukocyte activation and migration [78]. The air pouch model is a useful in vivo model for studying localized inflammation with no systemic effects. The injection of air subcutaneously into the thoracic area induces morphological alterations in the pouch’s cellular lining that lasts several days [39]. This structural change develops in pouch lining, similar to that which occurs in the synovial cavity. Carrageenan injection directly into the air cavity produces an inflammatory response in patients with rheumatoid arthritis and several other chronic inflammatory diseases. Therefore, this model can be employed to screen anti-inflammatory compounds [79]. One of the major benefits of air pouch inflammation over carrageenan injection directly into the knee joint is the increased volume of pouch exudate, allowing for the measurement of several parameters from each animal [80,81]. Peritonitis induced by carrageenan is a well-established model of acute inflammation frequently used for testing novel anti-inflammatory drugs focusing on analysis or quantification of cellular migration, vascular permeability, and measurement of inflammatory parameters [82]. In the current study, both the air pouch and carrageenan-induced peritonitis showed significant infiltration of leukocytes, neutrophils, and monocytes to inflammatory exudates. The lining of the air pouch was intensely invaded with inflammatory cells. Neutrophil migration to the joints of rheumatoid arthritis patients causes the destruction of synovial tissue, cartilage structure and bones through the release of different proteases and harmful oxygen metabolites. The administration of *T. foenum-graecum* extract efficiently abrogated the cellular influx to the pouch exudates and reduced the morphological changes in the lining of the pouch tissues.

The production of free radicals at the inflammatory site is one of the major mechanisms of tissue damage produced by several inflammatory disorders [11,12]. Activated neutrophil infiltration to the inflammatory sites is a significant source of proinflammatory mediators and oxygen-derived free radicals, which induces inflammatory reactions [83]. It has been shown that, after the injection of carrageenan, the level of free radicals, and thus total oxidant status (TOS), rises in both the air pouch and peritoneal exudates [84]. These free radicals may target the plasma membrane causing malondialdehyde (MDA) to accumulate. MDA is a basic marker of oxidative stress. Myeloperoxidase is an enzyme found in leucocytes, involved in the formation of a wide range of reactive oxygen species [85]. MPO-derived oxidants have been shown to lead to tissue damage during inflammation [42,43]. Tissue damage related to oxidative stress can be reversed via the CAT and SOD enzyme. The activity of these enzymes controls the cytotoxic properties of toxic free radicals [41,44]. In this study, there were significant increases in catalase and SOD enzyme activity in both the air pouch and peritoneal exudate; hence, there was an increase in TAS level after treatment with *T. foenum-graecum* extract. Furthermore, there was a substantial decrease in MDA and MPO levels after treatment with *T. foenum-graecum* extract. Oxidative stress may, therefore, be inhibited by hydroethanolic extract of *T. foenum-graecum*. A decrease in MDA and MPO activity is linked to reduced inflammatory progression, and these effects could be attributed to polyphenols, quercetin, and gallic acid, among other compounds found in the plant extract.

## 5. Conclusions

In conclusion, hydroethanolic extract of *T. foenum-graecum* contains a significant number of polyphenolic compounds, which decreases cellular infiltration, lipid peroxidation, and the level of pro-inflammatory cytokines and inflammatory mediators. Pre-treatment of animals with *T. foenum-graecum* extract significantly improves tissue antioxidant status, which ameliorates oxidative stress and inflammatory processes induced by carrageenan. Moreover, the *T. foenum-graecum* extract was also characterized through ESI-Q-TOF MS/MS as well as HPLC-PDA to investigate the phenolic profile likely responsible for bioactivity. However, further studies are essential and need to be conducted to investigate the mechanisms of action, bioaccessibility, and bioavailability of these compounds for their proper nutritional and medicinal properties.

## Figures and Tables

**Table 1:** High-pressure liquid chromatography equipped with diode array detector (HPLC-DAD) summary results of each peak.

Peak No.	Compound Name	Retention Time	ʎ_max_ (nm)	Concentration mg/100 g DW
1	Gallic acid (GA)	4.4	271.2, 214.6	117.6 ± 1.5
2	Trihydroxybenzoic (TBHA) acid	7.3	260.5, 221.4	112.6 ± 5.6
3	Protochateuic acid (PTA)	13.4	291.4, 260.5, 214.4	103.8 ± 2.4
4	Ferulic acid (FA)	14.1	323.7, 236.8	168.4 ± 1.8
5	Protocatechualdehyde (PTCA)	15.0	308.2, 279.5, 229.7	98.4 ± 2.3
6	p-coumaric acid (PCA)	18.4	309.4, 229.7	256.7 ± 6.8
7	Vanillic acid (VN)	19.7	279.5, 246.4	57.9 ± 2.4
8	Protocatechualdehyde (PhA)	22.2	280.6, 224.3	116.8 ± 1.9
9	Vaniline (VNA)	23.6		87.5 ± 2.4
10	Apigenic acid (APG)	26.9	294.6, 261.7, 219.8	117.7 ± 3.6
13	Syringic acid (peak 13)	27.2	310.5, 222.5	95.6 ± 11.3
14	p-hydroxybenzoic acid (PHBA)	28.1	328.5, 234.4	113.7 ± 3.8

**Table 2:** Ultra-high-pressure liquid chromatography equipped with electrospray ionization-quadrupole time-of-flight mass detector (UPLC-ESI-Q-TOF-MS/MS) characterization of phytochemicals of *T. foenum.*

Serial#	Compound Detected	Retention Time (min)	MS	M − H (Predicted)	M − H (Found)	MS2	Formula	Reference
1	Catechin 3-O-gallate	2.34	442.09	441.0827	441.0840	251.0365, 233.0297, 124.9848	C_22_H_18_O_10_	[49,50]
2	4-O-methylepigallocatechin	11.20	320.0896	321.1569	321.1468	303.1364, 241.1007	C_16_H_16_O_7_	[50,51,52]
3	Sativanone	12.84	300.1398	299.1425	299.1474	283.1462, 223.0883, 179.0974	C_17_H_16_O_5_	[50,52]
4	Gallic acid	13.36	170.12	169.1345	169.1342	125.4622	C_7_H_6_O_5_	[50,52]
5	Kampferol	18.34	286.23	285.1487	285.1425	245.1357 189.1109	C_15_H_10_O_6_	[50,53]
6	Methylviolanone	18.57	330.1103	329.1103	329.1539	311.1424, 279.1167	C_18_H_18_O_6_	[20,50]
7	homovanilic acid-hexoside	19.23	344.138	343.1135	343.1762	279.1162, 255.1146, 181.0796, 163.0396	C_15_H_20_O_9_	[50,51]
8	5-O-Feruloylquinic acid	20.99	368.1568	367.1448	367.1648	337.1599, 293.1295, 191.0640	C_17_H_20_O_9_	[49,50,52]
9	Cyanidin	25.17	288.2	287.2	287.1768	287.1768, 253.095, 167.1364, 149.1272	C_15_H_11_O_6_	[53,54]
10	Coumaroylquinic acid	26.09	338.1002	337.1927	337.1900	265.1338, 173.1554	C_16_H_18_O_8_	[51,52,53]
11	Quercetin	27.15	302.236	301.1915	301.1950	295.1747 291.1551, 163.1428	C_15_H_10_O_7_	[20,52]
12	Schisandrin C	27.49	384.1573	385.1646	385.1393	339.1704, 137.0888, 123.0786	C_22_H_24_O_6_	[20,52]
13	p-coumaric acid O-hexoside	27.95	326.1455	325.1529	325.1552	295.1080, 165.0856, 123.0763	C_15_H_18_O_8_	[20,52]
14	Hydroxytyrosol-4-o-glycoside	29.29	316.2158	315.2085	315.2140	251.1190, 237.1404, 237.1042 177.1555	C_14_H_20_O_8_	[50,55]
15	3-Methoxysinensetin	31.42	402.2315	403.2388	403.2755	399.2602, 383.2710, 199.0671	C_21_H_22_O_8_	[52,54]
16	Pinoresinol	31.74	358.1416	357.1343	357.2531	313.2297,	C_20_H_22_O_6_	[52,54]
17	Glycitin	32.45	446.3213	447.3286	447.3361	403.3065, 368.4185	C_22_H_22_O_10_	[20,52]
18	Dihydroferulic acid 4-O-glucuronide	33.52	372.1156	371.1054	371.1674	291.1162, 266.9912, 73.0421	C_6_H_20_O_10_	[50,55]

**Table 3:** In vitro antioxidant activities and total phenolic and flavonoid contents of *T. foenum-graecum* seed extract.

Antioxidant Assay	*T. foenum-graecum*	Ascorbic Acid
Total phenolic contents (GAE mg/g)	454.93 ± 3.57	-
Total flavonoid contents (CE µg/g)	135.04 ± 2.12	-
Total antioxidant capacity (AAE mg/g)	162.51 ± 3.81	-
DPPH inhibition assay (IC_50_) (µg/mL)	24.7 ± 2.70 ^a^	25.05 ± 1.45 ^a^
ABTS cation inhibition (IC_50_) (µg/mL)	15.8 ± 0.87 ^a^	-

Each value is mean ± SD of three replicates. Alphabets a shows significant difference at (*p* < 0.05). -, not determined; AAE, ascorbic acid equivalents; CE, catechin equivalents; GAE, gallic acid equivalents; (µg/mL), microgram per milliliter.

**Table 4:** Sign and symptoms of acute oral toxicity in rats after treatment with *T. foenum-graecum* seeds extract.

Observation	Control Group	Treatment Group
Behavior change	NO	NO
Salivation	NO	NO
Eye/skin appearance	NC	NC
Sleep	NO	NO
Diarrhea	NO	NO
Lethargy	NO	NO
Coma	NO	NO
Respiration/cardiac output	NO	NO
Mortality	NO	NO

**Table 5:** Effect of *T. foenum-graecum* seed extracts on body weights (gram) of male and female rats.

Sex	Treatment	Time				
		Day 1	Day 7	Day 14	Day 21	Day 28
Male	Control	213.78 ± 3.39	223.54 ± 2.38	240.12 ± 5.79	272.13 ± 1.36	310.13 ± 1.05
	*T. foenum-graecum*	210.14 ± 1.68	223.67 ± 2.30	242.27 ± 1.88 ^c^	265.55 ± 1.25 ^c^	295.53 ± 1.14 ^c^
Female	Control	215.12 ± 1.05	230.59 ± 1.21	239.16 ± 2.68	290.19 ± 3.39	319.12 ± 5.81
	*T. foenum-graecum*	212.1 ± 2.18 ^b^	211.66 ± 4.99 ^a^	233.12 ± 1.55 ^c^	250.41 ± 2.94 ^c^	269.64 ± 3.14 ^c^

Each value is mean ± SD of three replicates; values with superscripts a, b and c are significant at 0.05, 0.01 and 0.001.

**Table 6:** Relative weight of organs (g/g body weight) after treatment with *T. foenum-graecum* seed extract for 28 days.

Sex	Organ	Control	*T. foenum-graecum*
Male	Liver	2.46 ± 0.32	2.51 ± 0.47
	Kidney	0.49 ± 0.03	0.49 ± 0.05
Female	Liver	2.09 ± 0.16	2.31 ± 0.08
	Kidney	0.49 ± 0.11	0.46 ± 0.30

Values are presented as mean ± SD.

**Table 7:** Effect on hematological parameters in male and female rats after treatment with *T. foenum-graecum* seed extract.

Parameter	Male		Female	
	Control	*T. foenum-graecum*	Control	*T. foenum-graecum*
Hemoglobin (g/dL)	14.0 ± 1.40	14.0 ± 1.83	14.0 ± 1.5	15.0 ± 0.73
Packed cell volume	42 ± 1.05	43 ± 1.11	42 ± 1.68	46 ± 2.55
Red Blood Cells (10^6^/µL)	7.7 ± 0.35	7.4 ± 0.53	6.68 ± 0.15	7.2 ± 0.19
Mean corpuscular volume (fL)	52 ± 1.13	54 ± 1.31	57 ± 2.62	59 ± 1.64
Mean cell hemoglobin (pg)	22 ± 2.54	21 ± 1.41	19.59 ± 2.23	19.91 ± 2.54
Mean corpuscular hemoglobin concentration (g/L)	35 ± 1.72	34 ± 1.03	36.01 ± 1.99	33.08 ± 1.88
White blood cells (10^9^/L)	9.0 ± 0.31	8.9 ± 0.54 ^b^	7.90 ± 0.93	6.1 ± 0.26 ^b^
Platelets (10^9^/L)	161 ± 2.43 ^c^	216 ± 3.14 ^c^	160 ± 11.46	225 ± 16.42 ^c^
Neutrophils (%)	10 ± 0.36	9 ± 0.52	10 ± 0.22	11 ± 0.41
Lymphocytes (%)	82 ± 2.74	85 ± 2.64 ^a^	82 ± 2.65	79 ± 2.95 ^a^
Monocytes (%)	6 ± 0.04	4 ± 0.53 ^b^	5 ± 0.62	7 ± 0.17
Eosinophils (%)	2 ± 0.08	2 ± 0.68 ^b^	3 ± 0.81	3 ± 0.19

Each value is mean ± SD of three replicates; values with superscripts a, b and c are significant at 0.05, 0.01 and 0.001.

**Table 8:** Effect on biochemical parameters in male and female rats after treatment with *T. foenum-graecum* seed extract.

Parameter	Male		Female	
	Control	*T. foenum-graecum*	Control	*T. foenum-graecum*
Aspartate aminotransferase (AST) (U/L)	34 ± 0.97	21 ± 2.04 ^c^	30 ± 1.66	21 ± 1.53 ^c^
Alanine aminotransferase (ALT) (U/L)	35 ± 1.23	24 ± 1.32 ^b^	33 ± 1.50	25 ± 1.71 ^a^
Alkaline phosphatase (ALP) (U/L)	449 ± 2.41	447 ± 1.78 ^c^	452 ± 2.72	442 ± 1.24 ^c^
Gamma Glutamyl Transferase (γ-GT) (U/L)	6.16 ± 0.56	5.70 ± 0.72 ^a^	6.71 ± 0.77	4.72 ± 0.15 ^c^
Total Bilirubin (μmol/L)	0.6 ± 0.03	0.6 ± 0.01 ^b^	0.6 ± 0.16	0.6 ± 0.16
Direct Bilirubin (μmol/L)	0.2 ± 0.04	0.3 ± 0.03	0.2 ± 0.04	0.2 ± 0.06
Indirect Bilirubin (μmol/L)	0.4 ± 0.02	0.3 ± 0.07	0.4 ± 0.05	0.4 ± 0.09
Glucose(mmol/L)	4.61 ± 1.56	4.93 ± 0.87	4.88 ± 1.00	4.72 ± 0.11
Creatinine (μmol/L)	60.27 ± 1.09	59.17 ± 1.90	61.16 ± 5.17	55.25 ± 2.26
Total cholesterol (mmol/L)	1.69 ± 0.32	1.47 ± 0.07	1.77 ± 0.52	1.41 ± 0.40
Triglycerides (mmol/L)	0.84 ± 0.37	0.80 ± 0.14	0.90 ± 0.15	0.84 ± 0.11
Total protein (g/L)	4.01 ± 0.59	3.91 ± 0.62	4.39 ± 0.45	4.28 ± 0.49
Blood Urea Nitrogen (mmol/L)	8.13 ± 0.37	7.08 ± 1.18 ^a^	9.11 ± 0.71	6.54 ± 1.16 ^a^

Each value is mean ± SD of three replicates; values with superscripts a, b and c are significant at 0.05, 0.01 and 0.001.

**Table 9:** Effects of different doses of *T. foenum-graecum* seed extract on oxidative stress and lipid peroxidation in air pouch exudate.

Treatment	Dose (mg/kg)	Myeloperoxidase (U/mg Protein)	Malondialdehyde (nmol/mg Protein)	Catalase (µmol H_2_O_2_/µg of Protein)	Superoxide Dismutase (U/mg of Protein)	Total Oxidant Status (µM H_2_O_2_ Eq/g)	Total Antioxidant Status (µM Trolox Eq/g)
Control	-	364 ± 1.54	5.84 ± 4.61	321.37 ± 2.87	4.39 ± 0.28	1.98 ± 0.45	6.35 ± 0.28
Carrageenan	-	647 ± 1.46 ^c^	14.46 ± 1.67 ^c^	218.38 ± 4.53	2.39 ± 0.21	13.21 ± 0.48 ^c^	0.28 ± 0.05
*T. foenum-graecum*	100	273 ± 2.01	13.28 ± 1.72	328.48 ± 2.23	4.39 ± 0.38 ^b^	9.98 ± 1.16	6.39 ± 0.60 ^a^
	200	214 ± 1.03	9.99 ± 1.44	438.38 ± 3.99	5.92 ± 0.83	7.95 ± 0.71	6.54 ± 0.75
	400	184 ± 2.04	7.98 ± 1.14	647.29 ± 8.31 ^c^	6.20 ± 0.21 ^c^	6.47 ± 1.39	8.84 ± 1.25 ^b^
Dexamethasone	20	173 ± 1.27 ^a^	8.30 ± 1.87 ^a^	438.58 ± 11.60 ^b^	7.31 ± 0.33	0.27 ± 0.32	13.68 ±1.99 ^c^

Each value is mean ± SD of three replicates; values with superscripts a, b and c are significant at 0.05, 0.01 and 0.001.

**Table 10:** Effects of different doses of *T. foenum-graecum* seed extract on oxidative stress and lipid peroxidation in carrageenan-induced peritonitis.

Treatment	Dose (mg/kg)	Myeloperoxidase (U/mg Protein)	Malondialdehyde (nmol/mg Protein)	Catalase (µmol H_2_O_2_/µg of Protein)	Superoxide Dismutase (U/mg of Protein)	Total Oxidant Status (µM H_2_O_2_ Eq/g)	Total Antioxidant Status (µM Trolox Eq/g)
Control	-	34 ± 0.24	1.45 ± 0.01	212.07 ± 2.97	1.39 ± 0.08	0.85 ± 0.01	1.65 ± 0.28
Carrageenan	-	177 ± 3.06	4.86 ± 0.27	109.48 ± 0.43	0.89 ± 0.16	0.22 ± 0.08	0.61 ± 0.05
*T. foenum-graecum*	100	31.42 ± 0.61	4.38 ± 0.21 ^b^	108.08 ± 0.13 ^a^	1.12 ± 0.81 ^a^	0.48 ± 0.06	1.09 ± 0.20
	200	29.09 ± 0.23	3.99 ± 0.14	148.58 ± 0.69	1.16 ± 0.38	0.61 ± 0.01	1.54 ± 0.19
	400	18.53 ± 0.41	2.98 ± 1.14	205.29 ± 1.61 ^c^	1.21 ± 0.19 ^b^	0.98 ± 0.04 ^a^	1.49 ± 0.12
Dexamethasone	20	15.98 ± 108.27 ^c^	1.10 ± 0.07 ^c^	201.18 ± 1.46	1.46 ± 0.35	0.97 ± 0.02	1.88 ± 0.67 ^b^

Each value is mean ± SD of three replicates; values with superscripts a, b and c are significant at 0.05, 0.01 and 0.001. Eq: Equivalent.

## Data Availability

Data is contained within the article and Supplementary Materials.

## Supplementary Materials

The following supporting information can be downloaded at: https://www.mdpi.com/article/10.3390/antiox11020364/s1, Figure S1: MS/MS spectrum of *T. foenum* at retention time 2.341 min; Figure S2: MS/MS spectrum of *T. foenum* at retention time 11.199 min; Figure S3: MS/MS spectrum of *T. foenum* at retention time 13.359 min; Figure S4: MS/MS spectrum of *T. foenum* at retention time 18.123 min; Figure S5: MS/MS spectrum of *T. foenum* at retention time 18.123 min; Figure S6: MS/MS spectrum of *T. foenum* at retention time 18.574 min; Figure S7: MS/MS spectrum of *T. foenum* at retention time 19.233 min; Figure S8: MS/MS spectrum of *T. foenum* at retention time 20.989 min; Figure S9: MS/MS spectrum of *T. foenum* at retention time 25.107 min; Figure S10: MS/MS spectrum of *T. foenum* at retention time 26.096 min; Figure S11: MS/MS spectrum of *T. foenum* at retention time 27.155 min; Figure S12: MS/MS spectrum of *T. foenum* at retention time 27.950 min; Figure S13: MS/MS spectrum of *T. foenum* at retention time 29.299 min; Figure S14: MS/MS spectrum of *T. foenum* at retention time 31.424 min; Figure S15: MS/MS spectrum of *T. foenum* at retention time 31.742 min; Figure S16: MS/MS spectrum of *T. foenum* at retention time 32.456 min; Figure S17: MS/MS spectrum of *T. foenum* at retention time 33.518 min.
