# Supplementary material for: Chemical Fingerprinting, Antioxidant, and Anti-Inflammatory Potential of Hydroethanolic Extract of Trigonella foenum-graecum
Source: Antioxidants (Basel). 2022 Feb 11;11(2):364. doi: 10.3390/antiox11020364 (PMC8869320; doi:10.3390/antiox11020364)
Supplement: Supplementary file 1 [file antioxidants-11-00364-s001.zip › antioxidants-1562213-supplementary.pdf]

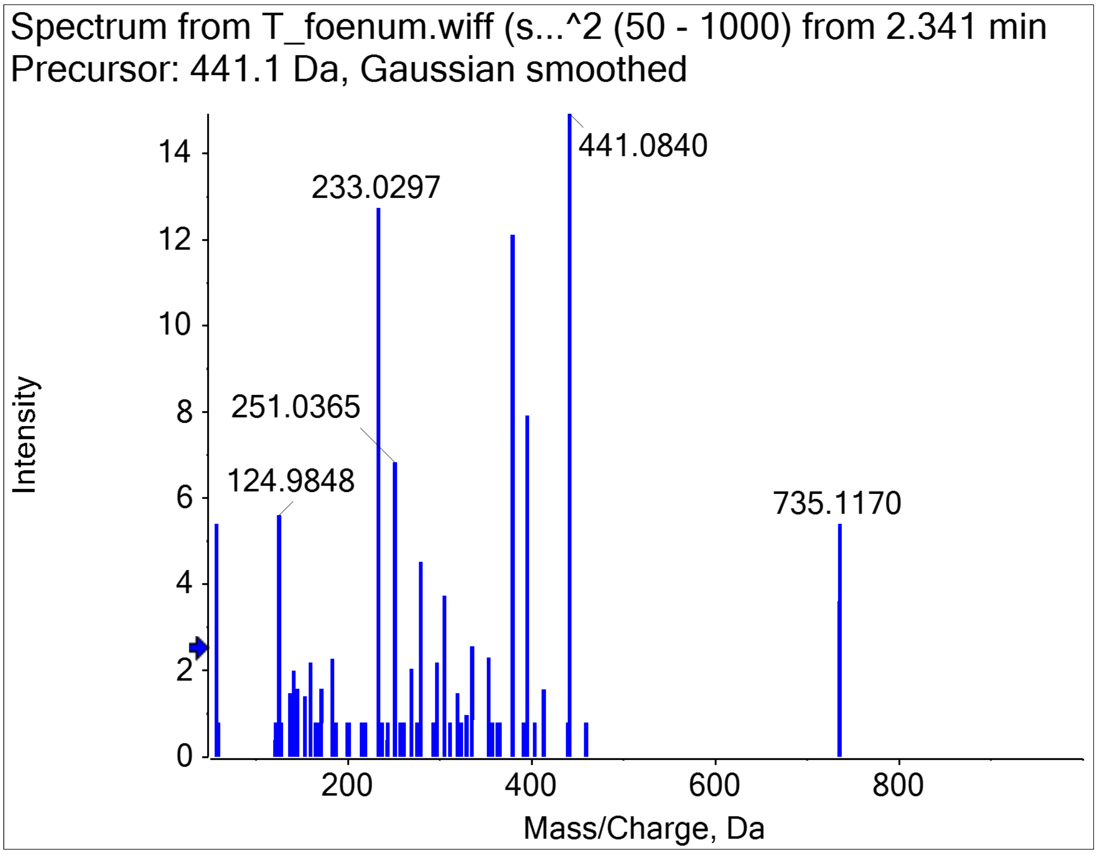


Figure S1: MS/MS spectrum of *T. foenum* at retention time 2.341 minute

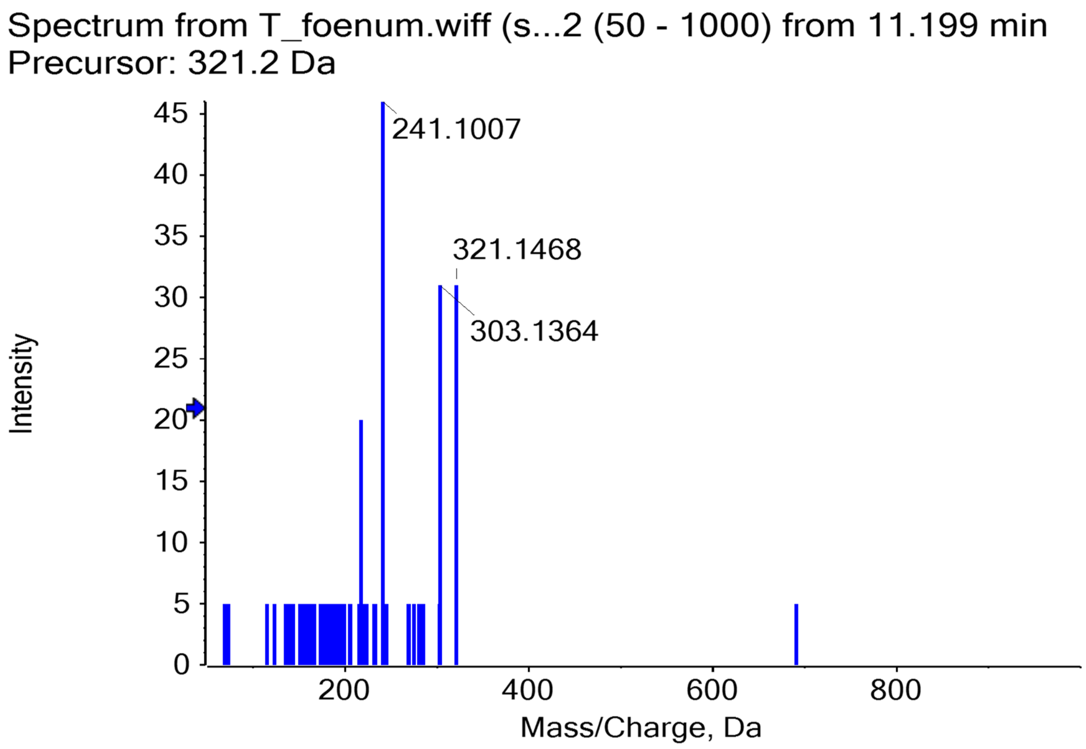


Figure S2: MS/MS spectrum of *T. foenum* at retention time 11.199 minute

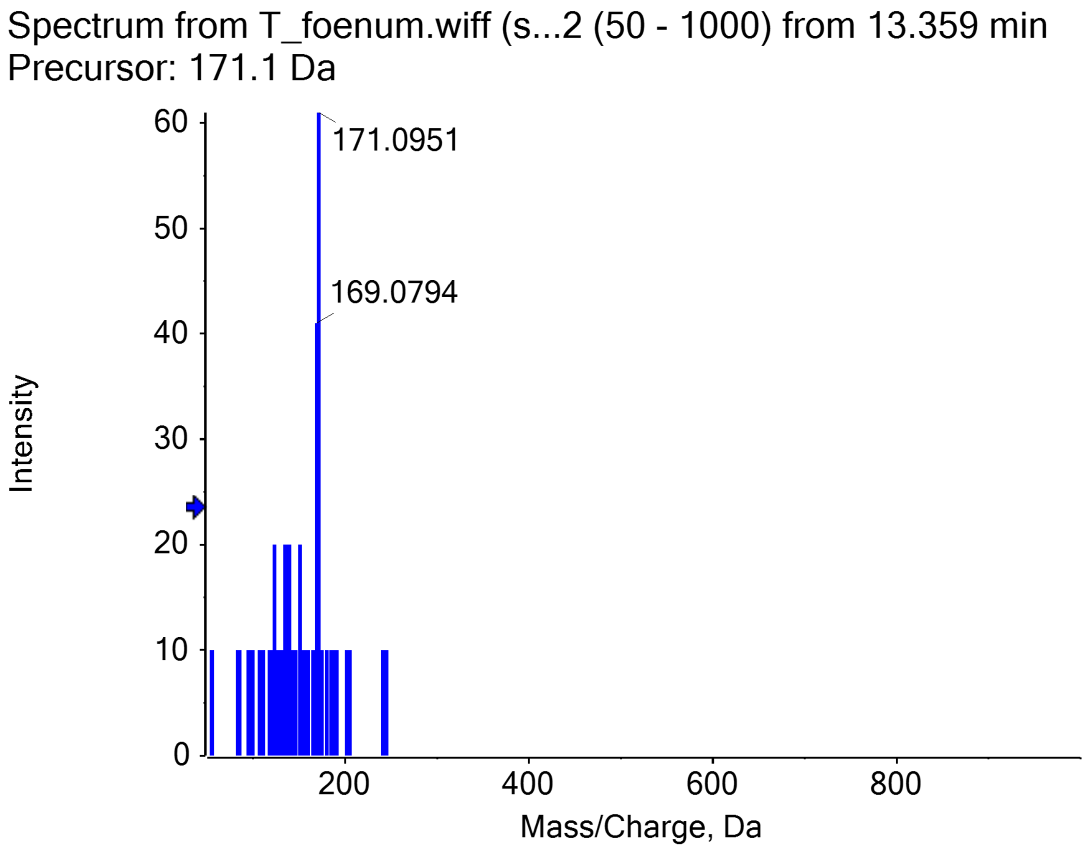


Figure S3: MS/MS spectrum of *T. foenum* at retention time 13.359 minute

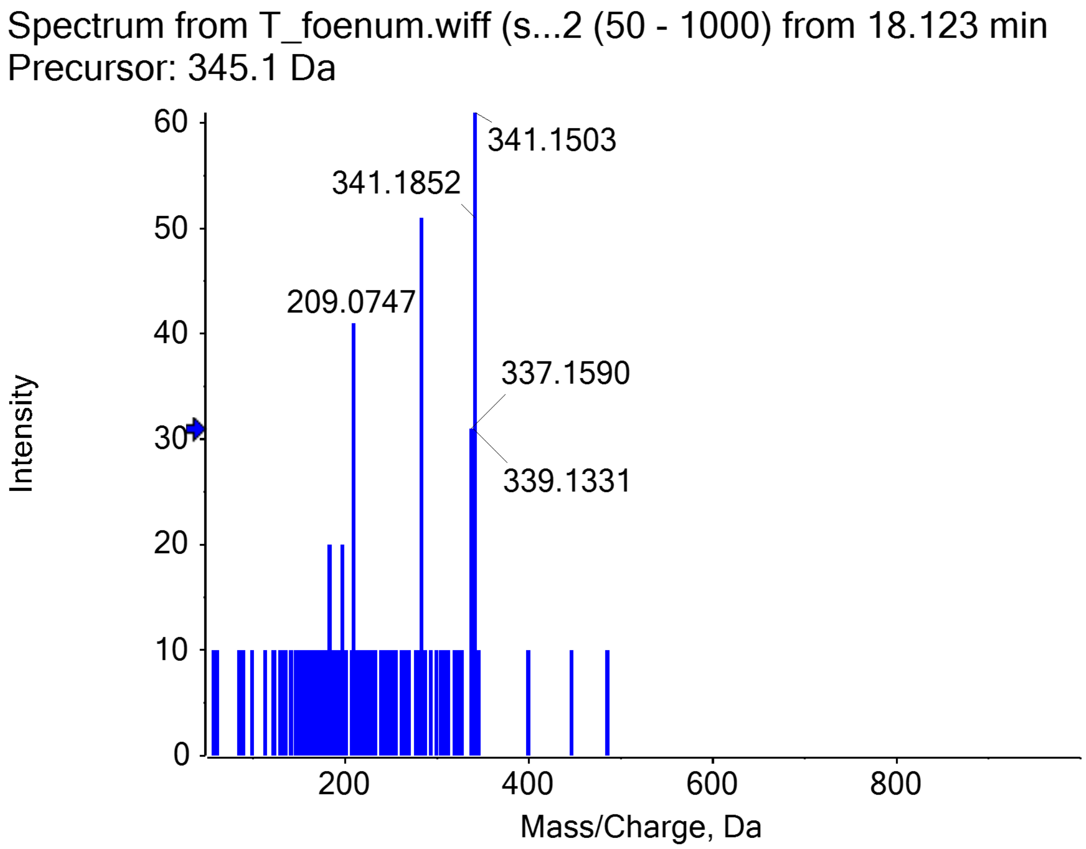


Figure S4: MS/MS spectrum of *T. foenum* at retention time 18.123 minute

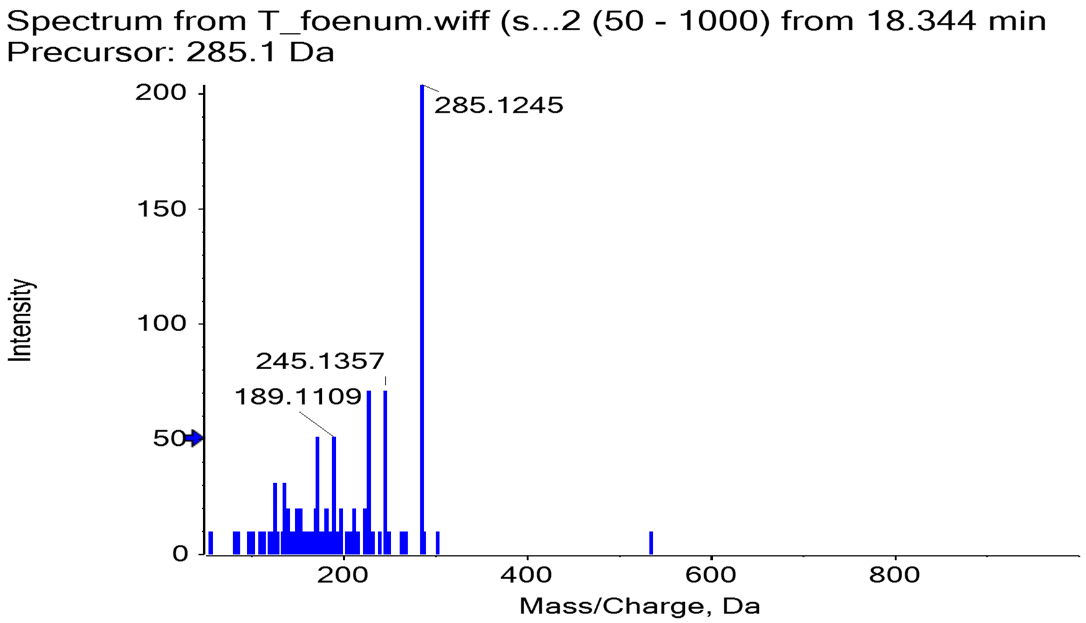


Figure S5: MS/MS spectrum of *T. foenum* at retention time 18.123 minute

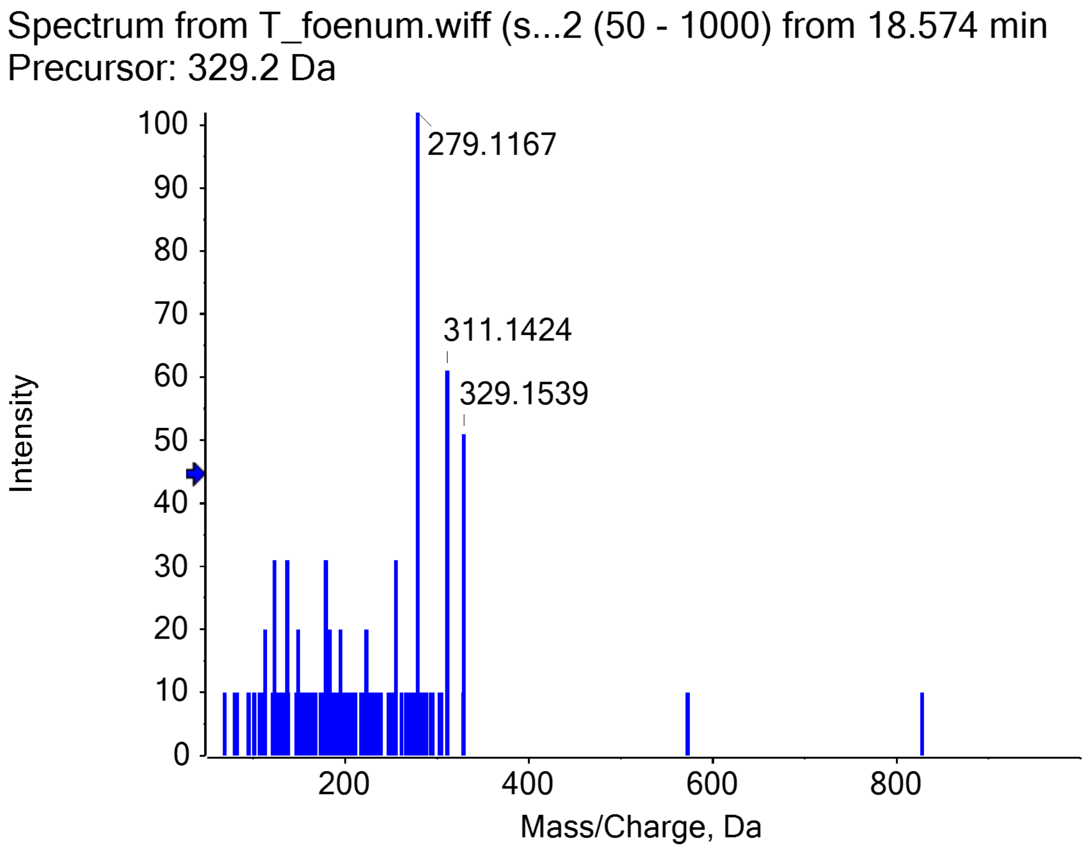


Figure S6: MS/MS spectrum of *T. foenum* at retention time 18.574 minute

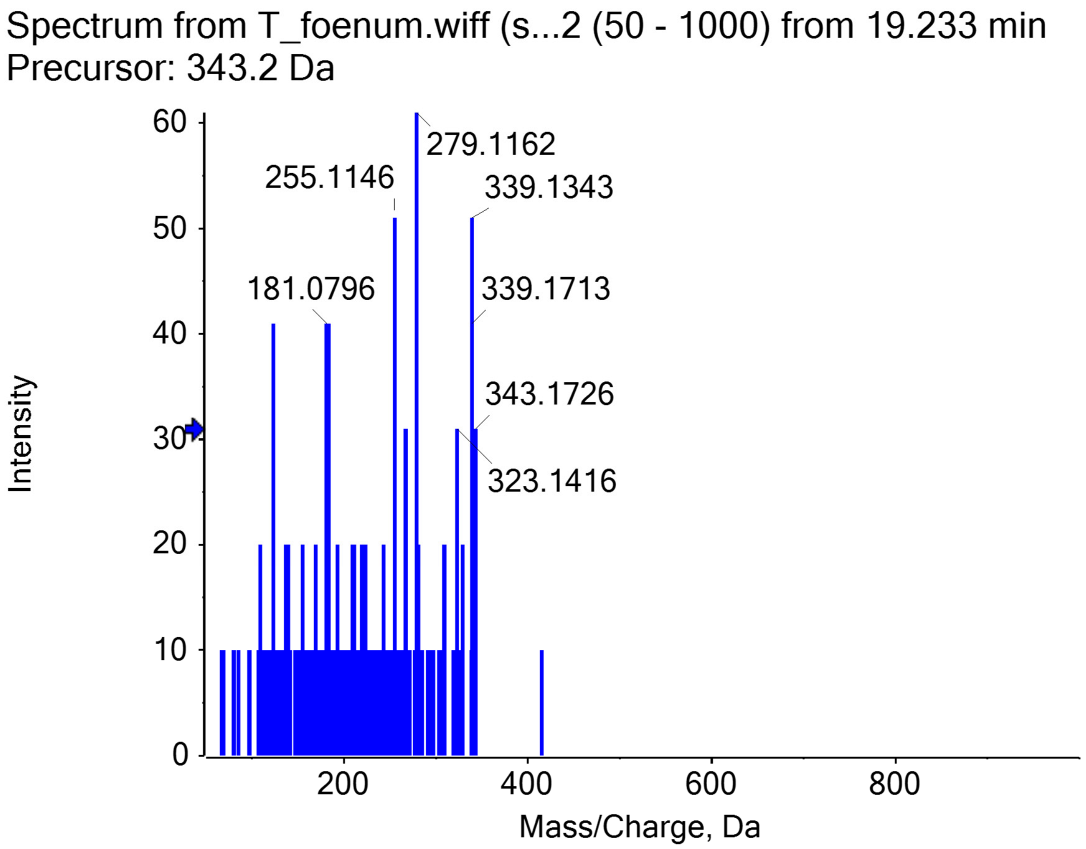


Figure S7: MS/MS spectrum of *T. foenum* at retention time 19.233 minute

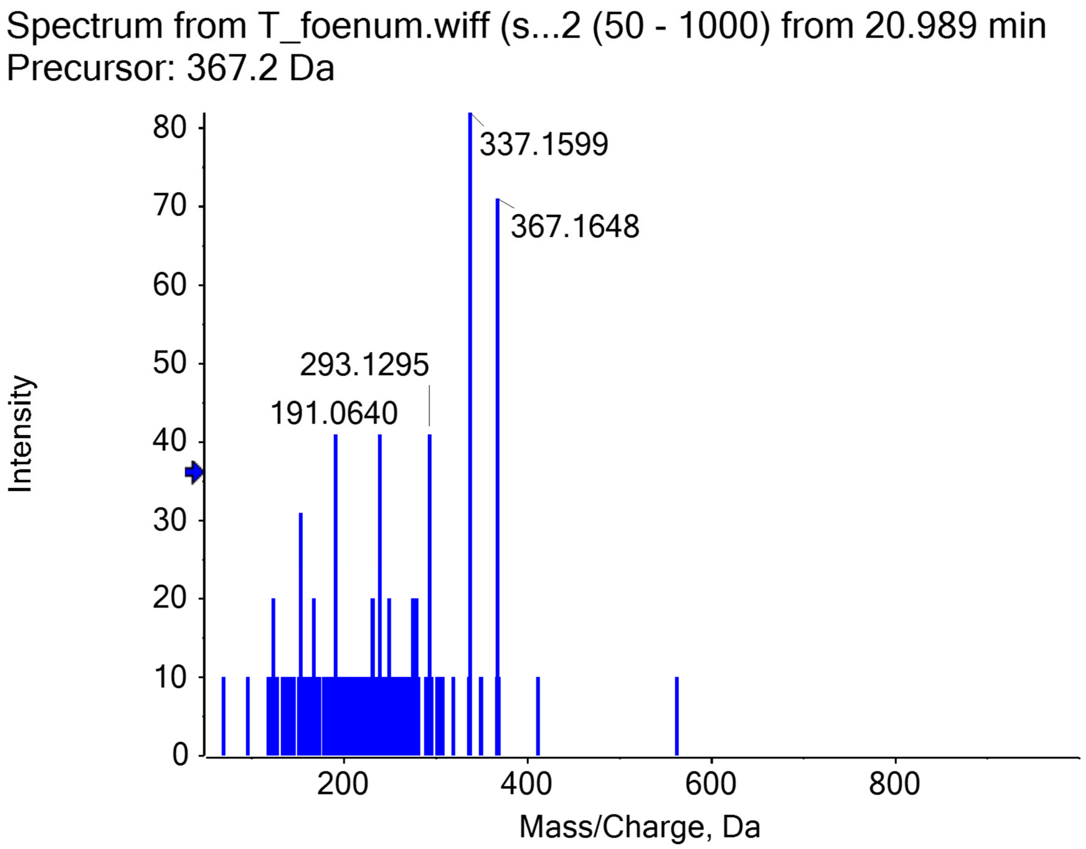


Figure S8: MS/MS spectrum of *T. foenum* at retention time 20.989 minute

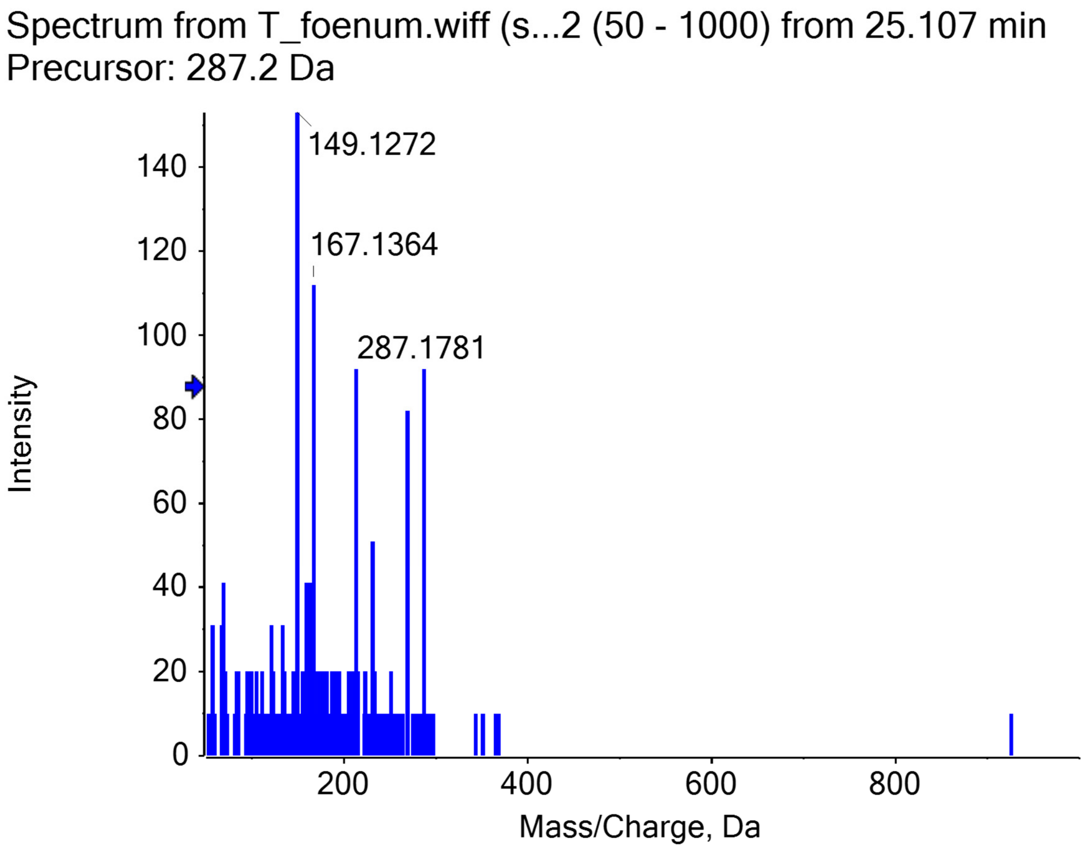


Figure S9: MS/MS spectrum of *T. foenum* at retention time 25.107 minute

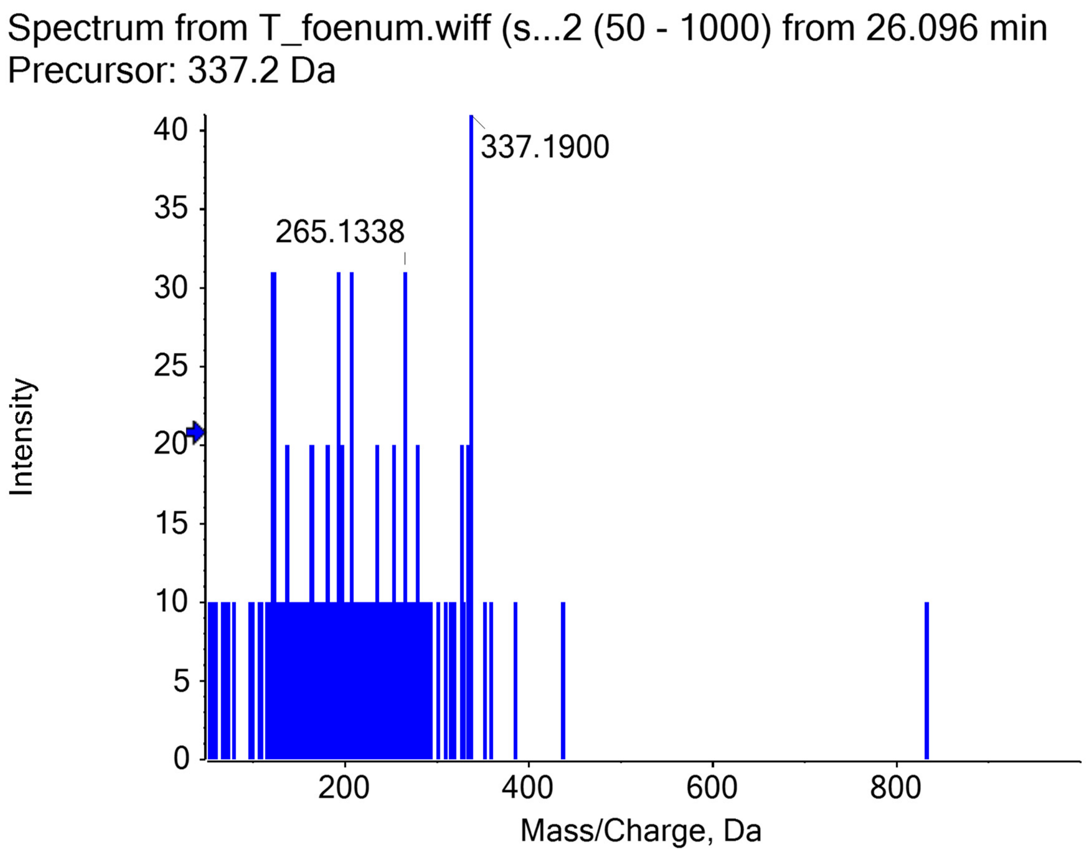


Figure S10: MS/MS spectrum of *T. foenum* at retention time 26.096 minute

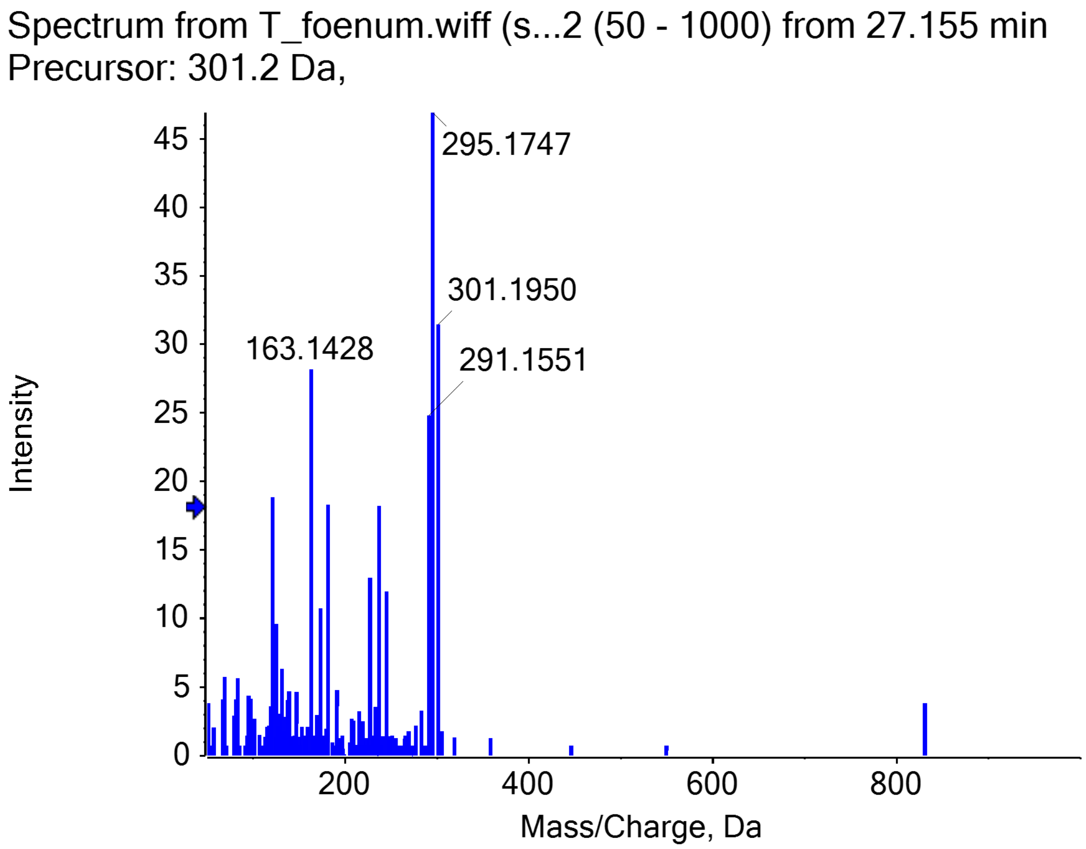


Figure S11: MS/MS spectrum of *T. foenum* at retention time 27.155 minute

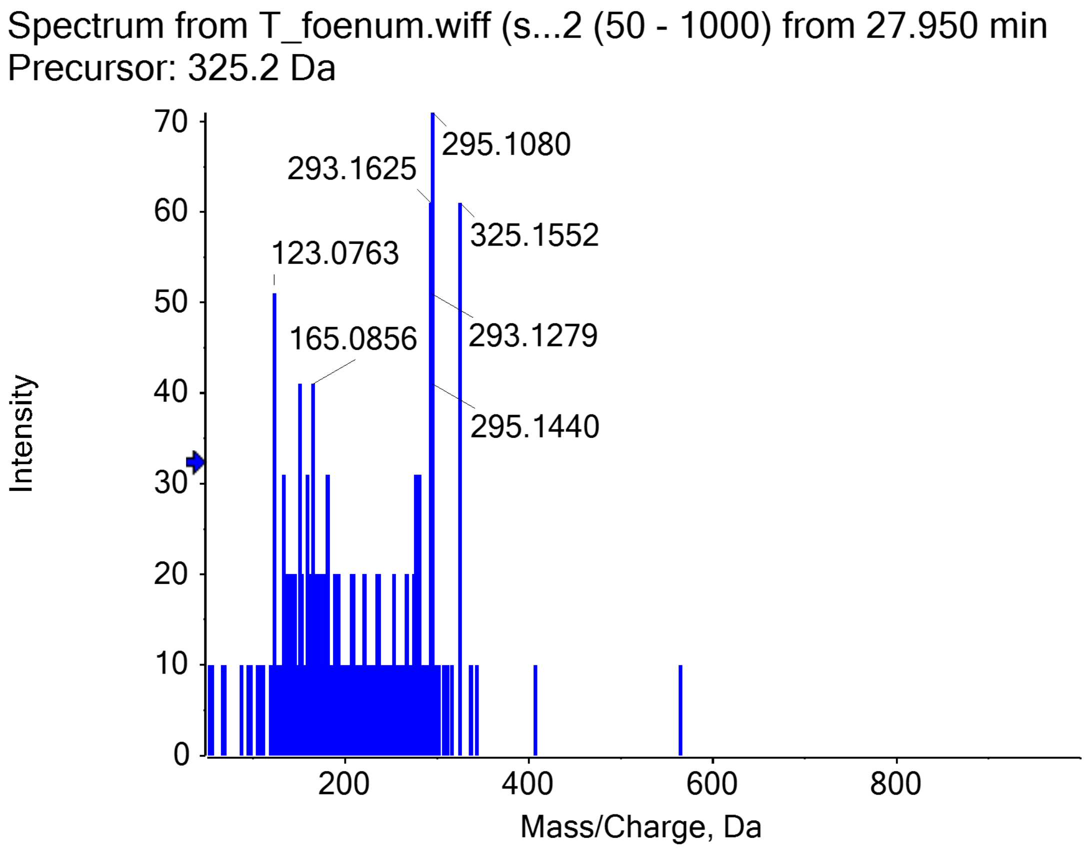


Figure S12: MS/MS spectrum of *T. foenum* at retention time 27.950 minute

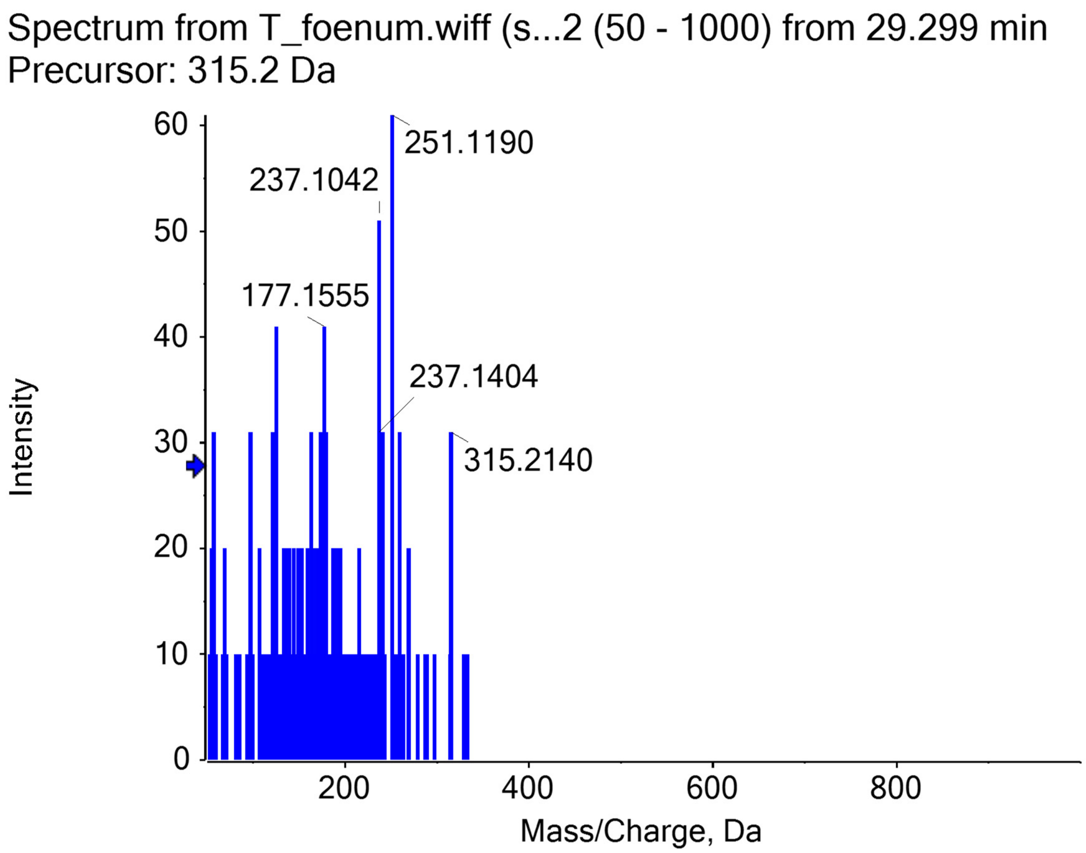


Figure S13: MS/MS spectrum of *T. foenum* at retention time 29.299 minute

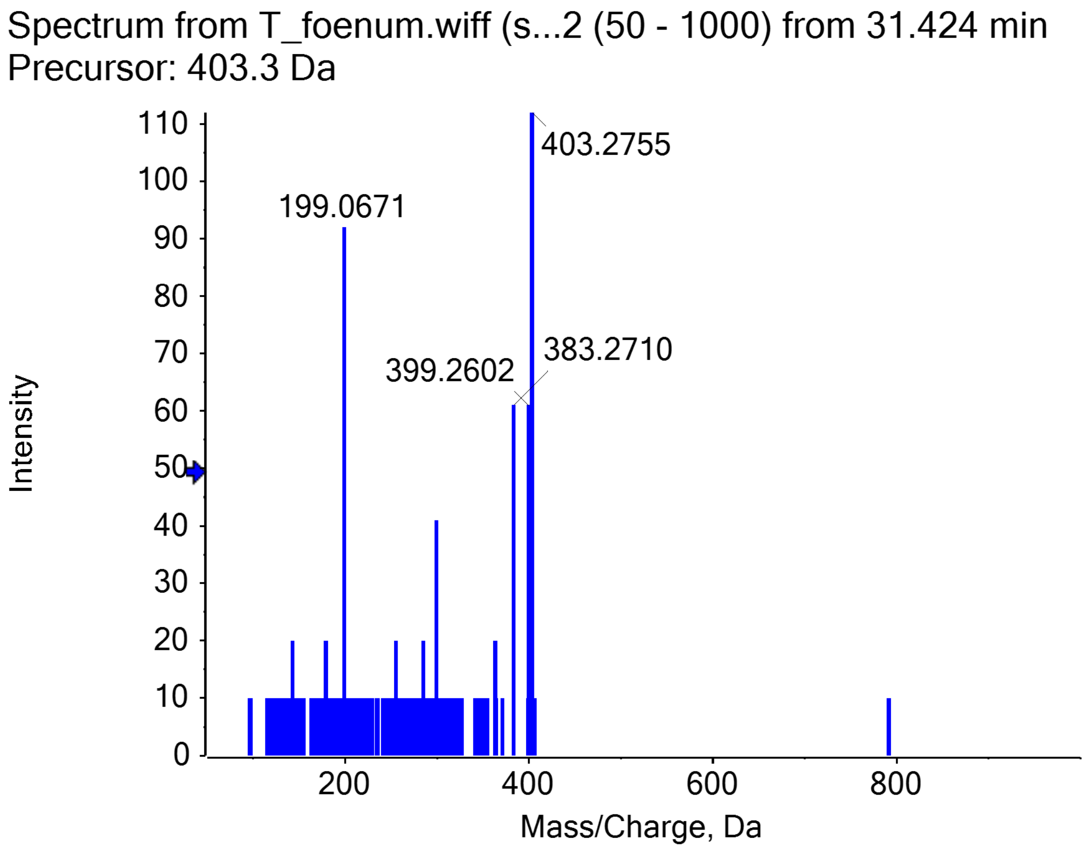


Figure S14: MS/MS spectrum of *T. foenum* at retention time 31.424 minute

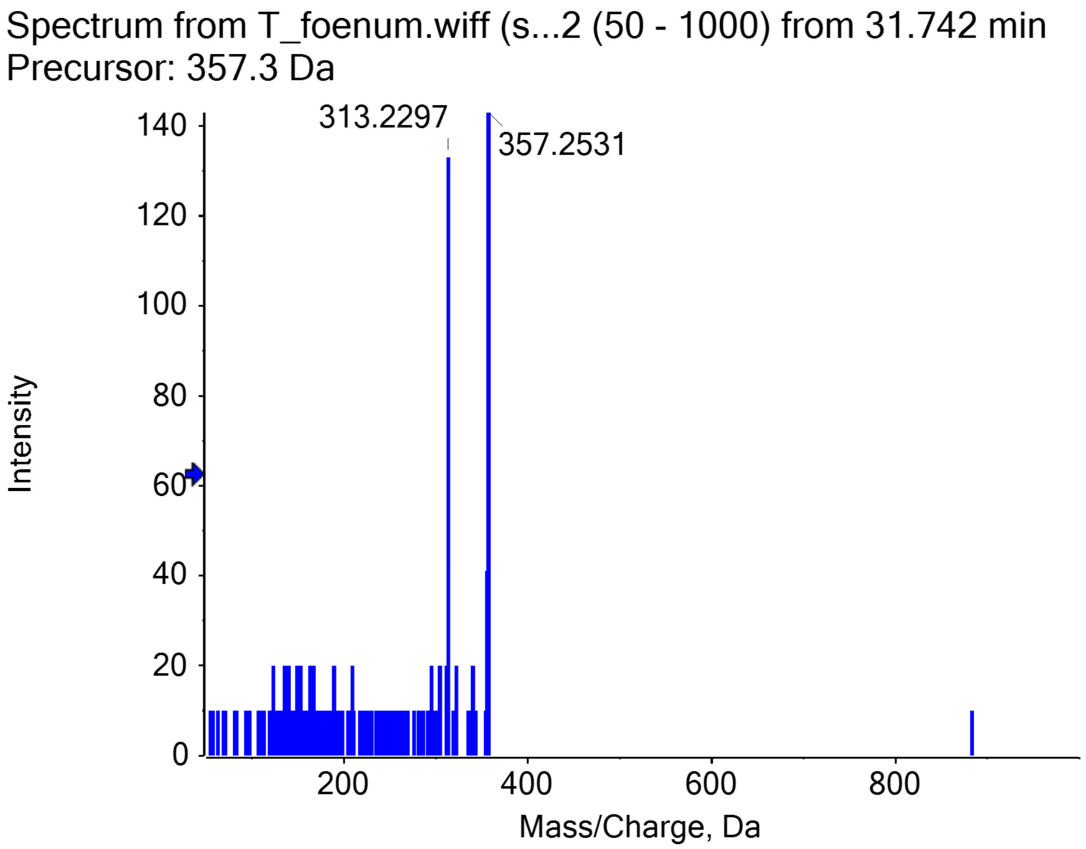


Figure S15: MS/MS spectrum of *T. foenum* at retention time 31.742 minute

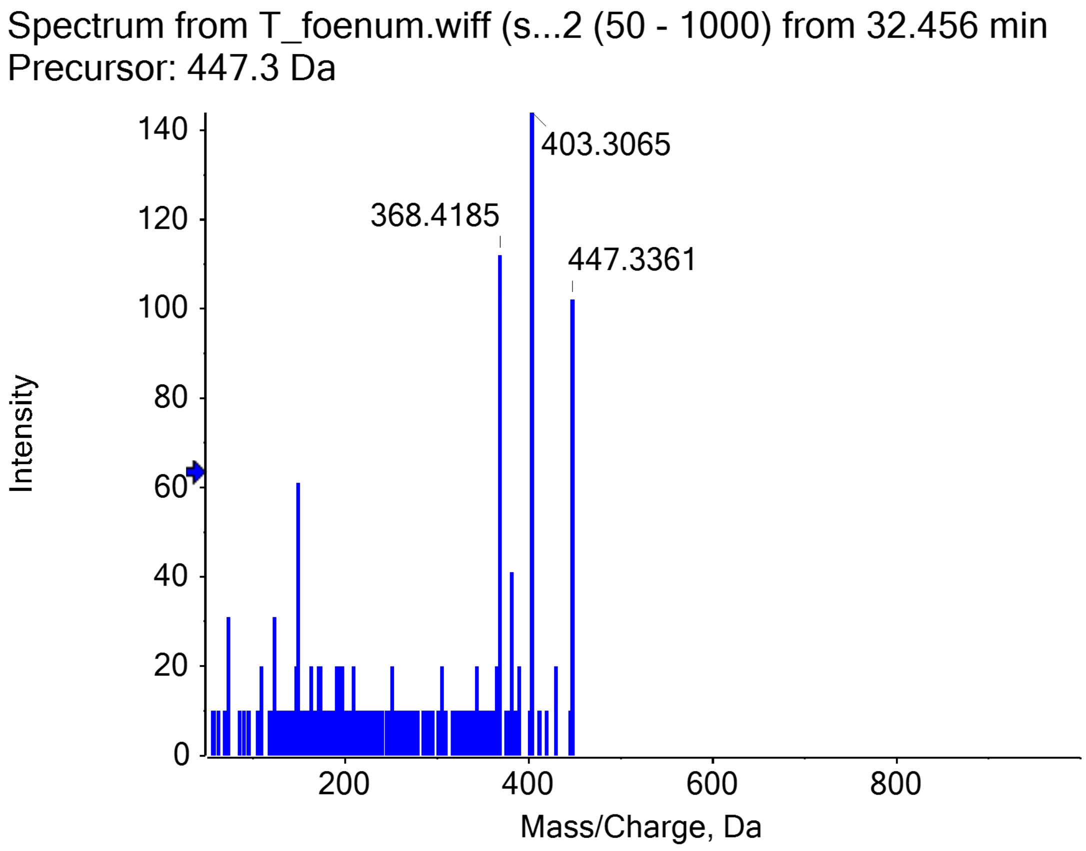


Figure S16: MS/MS spectrum of *T. foenum* at retention time 32.456 minute

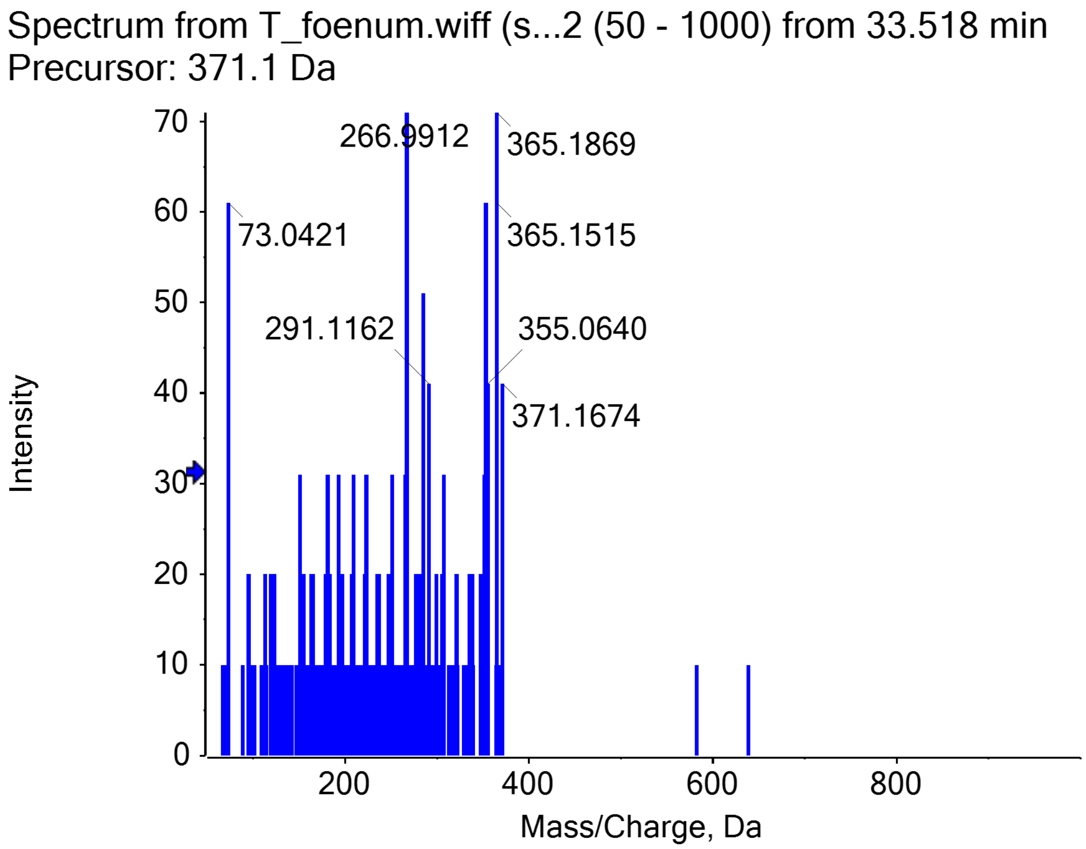


Figure S17: MS/MS spectrum of *T. foenum* at retention time 33.518 minute
